# Combined strategies with PARP inhibitors for the treatment of BRCA wide type cancer

**DOI:** 10.3389/fonc.2024.1441222

**Published:** 2024-08-02

**Authors:** Yijun Xie, Di Xiao, Duo Li, Mei Peng, Wei Peng, Huaxin Duan, Xiaoping Yang

**Affiliations:** ^1^ Department of Oncology, Hunan Provincial People’s Hospital, The First Affiliated Hospital of Hunan Normal University, Hunan Normal University, Changsha, Hunan, China; ^2^ Key Laboratory of Study and Discovery of Small Targeted Molecules of Hunan Province, Hunan Normal University, Changsha, Hunan, China; ^3^ Engineering Research Center of Reproduction and Translational Medicine of Hunan Province, Hunan Normal University, Changsha, Hunan, China; ^4^ Key Laboratory of Chemical Biology & Traditional Chinese Medicine Research of Ministry of Education, Hunan Normal University, Changsha, Hunan, China; ^5^ Key Laboratory of Protein Chemistry and Developmental Biology of Fish of Ministry of Education, Hunan Normal University, Changsha, Hunan, China; ^6^ Department of Pharmacy, Hunan Normal University, Changsha, Hunan, China; ^7^ School of Medicine, Hunan Normal University, Changsha, Hunan, China

**Keywords:** PARPi, combination therapy, homologous recombination, BRCA wild type, DNA single-strand breaks

## Abstract

Genomic instability stands out as a pivotal hallmark of cancer, and PARP inhibitors (PARPi) emerging as a groundbreaking class of targeted therapy drugs meticulously crafted to inhibit the repair of DNA single-strand breaks(SSB) in tumor cells. Currently, PARPi have been approved for the treatment of ovarian cancer, pancreatic cancer, breast cancer, and prostate cancer characterized by homologous recombination(HR) repair deficiencies due to mutations in BRCA1/2 or other DNA repair associated genes and acquiring the designation of breakthrough therapy. Nonetheless, PARPi exhibit limited efficacy in the majority of HR-proficient *BRCA1/2* wild-type cancers. At present, the synergistic approach of combining PARPi with agents that induce HR defects, or with chemotherapy and radiotherapy to induce substantial DNA damage, significantly enhances the efficacy of PARPi in *BRCA* wild-type or HR-proficient patients, supporting extension the use of PARPi in HR proficient patients. Therefore, we have summarized the effects and mechanisms of the combined use of drugs with PARPi, including the combination of PARPi with HR defect-inducing drugs such as ATRi, CHKi, HR indirectly inducing drugs like VEGFRi, CDKi, immune checkpoint inhibitors and drugs instigating DNA damage such as chemotherapy or radiotherapy. In addition, this review discusses several ongoing clinical trials aimed at analyzing the clinical application potential of these combined treatment strategies.

## 1 Introduction

Base excision repair (BER) is the primary mechanism for removing oxidized bases producing single strand break (SSB) intermediates. PARP1 (and PARP2) recognizes SSB and gaps in the DNA and catalyzes the addition of chains of ADP-ribose molecules to proteins in a process known as poly-ADP-ribosylation (PARylation) (1). PAR facilitates BER/SSBR by recruiting DNA repair factors and chromatin remodellers. PARP1/2 not only plays a crucial role in the repair of SSB (2). When PARP1/2 is deleted or inhibited, SSB accumulates and encounters replication forks during proliferation, which converts to DNA double-strand damage (DSB) (3). DSB is the most toxic DNA damage in cells, which is mainly repaired by high-fidelity HR pathway (4). When HR is deficient, DSB are primarily repaired through error-prone non-homologous end joining (NHEJ) pathway, and erroneous DNA repair can lead to genomic instability and cell death (5). Therefore, simultaneously inhibiting PARP1/2 and HR will lead cells to undergo synthetic lethality. Synthetic lethality refers to the phenomenon that the simultaneous inactivation of two genes will lead to cell or individual death, while the cell or individual can survive normally when either gene is inactivated alone. The application of the concept was first validated clinically in cancers with *BRCA1* and *BRCA2* mutations leading to HR repair deficiency (6, 7). Subsequently, PARP inhibitors (PARPi) have developed rapidly in clinical trials for ovarian and breast cancers with *BRCA1/2* mutations and other HR gene defects. Currently, six oral clinical PARPi effectively inhibit the catalytic function of PARP1 and PARP2 by competing with NAD+ for binding to PARP1/2 (8). Olaparib, Rucaparib, Niraparib, and Talazoparib have been approved by the Food and Drug Administration (FDA) for ovarian, breast, pancreatic and prostate cancer patients with *BRCA* mutations (8). In addition to catalytic inhibition, most PARPi also possess the ability to trap PARP on DNA, thereby preventing PARP1 protein from undergoing PARylation modification and releasing from damaged sites (9, 10). The formation of PARP1-DNA complex, which is trapped on the DNA, hinders the progression of replication fork and promotes DSB formation (10), which is the major mechanism by which PARPi kills HR-deficient cancer cells (10, 11). PARPi have high trapping capacity and can effectively kill cells with HR defects.

**Figure 1:**
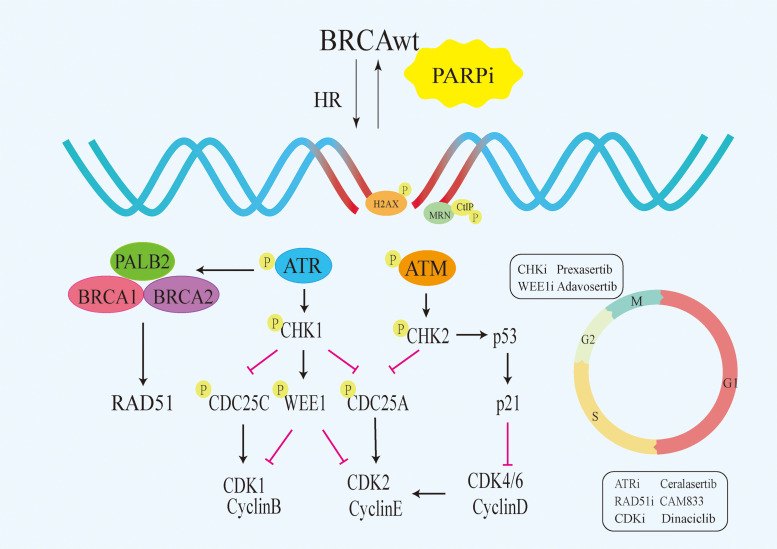
Signalling pathways that directly target HR. PARPi,PARP inhibitors;HR, homologous recombination;BRCAwt, BRCA wild-type;ATM, Ataxia Telangiectasia Mutated.

**Figure 2:**
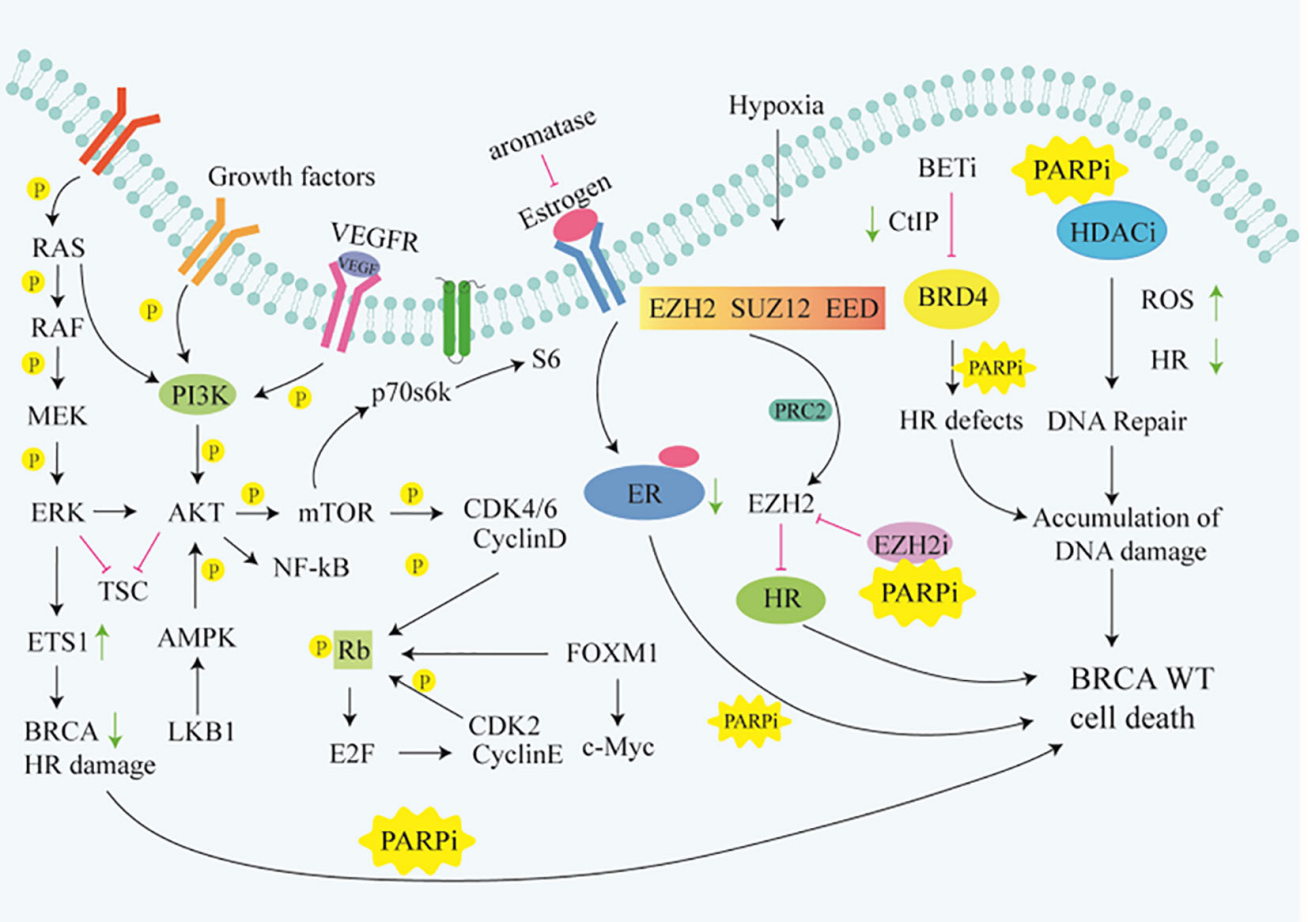
Signaling pathways that indirectly target HR. Rb, Retinoblastoma gene;ER, Retinoblastoma gene;TSC, Tuberous Sclerosis Complex; FOXM1,Forkhead box M1.

Although PARPi have achieved great success in the treatment of *BRCA* mutated patients, it has limited efficacy in *BRCA1/2* wild-type patients. According to research data, only about 20% of high-grade serous ovarian cancer patients who have *BRCA* mutations are more sensitive to PARPi, while the remaining approximately 80% of *BRCA* wild-type patients do not benefit from PARPi (12–14). Similarly, approximately 80% of triple-negative breast cancer (TNBC) patients without *BRCA* mutations do not respond to PARPi (15, 16). Due to the intact HR pathways in *BRCA* wild-type cells, even if PARPi prevent the repair of SSB and subsequent occurrence of DSB, tumor cells can still maintain chromosome stability and cell viability through HR (17). Consequently, the exploration of utilizing PARP inhibitors for treating *BRCA1/2* wild-type cancer patients has piqued the interest of researchers. Studies have revealed that, besides BRCA1/2, proteins such as MRN, ATM, CHK1, CtIP, RAD51 play direct roles in the HR process (**Figure 1**). Upon DNA damage, the MRE11A-NBS1-RAD50 (MRN), serves as a damage sensor by detecting DNA damage and binding to the break end (18). This complex then mediates the activation of ataxia telangiectasia mutated (ATM), which leads ATM to switch from an inactive dimer to an active monomer, phosphorylates sites at Ser-367, Ser-1893, Ser-1981, and Ser-2996 (19). These signals are relayed to CHK1 and BRCA1, while CtIP is ubiquitinated to facilitate S and G2 arrest (20, 21). Subsequently, RAD51 binds to the damaged DNA ends, forming DNA-RAD51 nucleoprotein filaments. At the same time, RAD51 can recognize a sister strand that matches the damaged DNA sequence, enabling recombination exchange between the two strands for DNA repair (22). Considering this, a basic strategy is to combine PARPi with drugs that may induce HR defects. For example, inhibiting ATR, CHK, and RAD51 to downregulate HR can enhance the sensitivity of *BRCA* wild-type patients to PARPi (23). Furthermore, the combination of PARP inhibitors with drugs that indirectly interfere with HR, such as VEGFR, BRD4, EZH2, HDAC, and PD-L1 inhibitors, results in synthetic lethality in *BRCA1/2* wild-type tumors, expanding the scope of PARP inhibitor applications (24–26) (**Figure 2**). Apart from HR inhibition, synergistic effects can be achieved by combining chemotherapy drugs or radiotherapy with PARP inhibitors, as those leads to further enhancement of DNA damage. In this review, we summarize the basic principles of combining PARP inhibitors in wild-type *BRCA* cancers, ongoing clinical trials, and analyze the future directions of PARPi combination therapy.

## 2 Inhibitors that directly suppress HR

### 2.1 ATR inhibitors

ATR (ataxia telangiectasia and Rad3-related) is a member of the Phosphoinositide 3-kinase (PIKK) family of serine/threonine protein kinases (27). ATR is a key regulatory factor of the DNA damage response (DDR) pathway, working together with other DDR proteins to initiate and coordinate the cell’s response to DNA damage and stress (28). Activated ATR kinase regulates various cellular processes, including inducing cell cycle arrest, replication initiation inhibition or restart and DSB repair (29–33). Multiple studies have identified BRCA1 as a target of ATR. BRCA1 expression is regulated by E2F, which in turn can be controlled by the ATR-CHK1 pathway (34–36). Prolonged chronic inhibition of ATR signaling depletes abundance of key HR factors such as BRCA1 and PALB2, significantly suppressing the cell’s ability to utilize HR-mediated DNA repair. Long-term treatment with ATR inhibitors(ATRi) can render HR-proficient cancer cells sensitive to PARPi (37). ATR promotes HR by phosphorylating PALB2 and enhances its repair of DNA damage by interacting with BRCA1 (38). Inhibition of ATR kinase aims to target tumor cells highly sensitive to high replication stress. While inhibiting ATR activity may induce replication fork stalling and collapse in normal cells, leading to some cytotoxicity, this cytotoxicity is further exacerbated in cancer cells with high replication stress (39–41). PARPi alone are insufficient to kill *BRCA* wild-type cancer cells, but their combination with ATRi shows synergistic inhibitory effects on cancer cells, leading to increased DNA damage (42). ATRi in combination with PARPi synergistically induce tumor cell death through inhibiting DNA repair pathways, leading to a synthetic lethality effect (43–45). Phosphorylation of Histone H2AX at Ser-139 is a marker of DNA damage. The ATR inhibitor Ceralasertib (AZD6738) can block CHK1 phosphorylation and increase γH2AX expression (46, 47). Preclinical studies have shown significant synergistic efficacy of Ceralasertib in combination with Olaparib in *BRCA* wild-type triple-negative breast cancer (TNBC) xenograft models, achieving complete tumor regression by increasing the dosage of Olaparib or Ceralasertib to twice daily (48).

Many patients develop resistance to PARPi, with acquired PARPi resistance being a major obstacle in treating tumors. A study on a patient-derived xenograft (PDX) model for platinum-resistant *BRCA* wild-type patients showed that the combination of ATRi and PARPi have a synergistic effect, leading to increased DNA damage and sustained regression of ovarian tumors, significantly improving patient survival (49). Currently, there is no definitive data on clinical trials of PARPi-ATRi therapy for *BRCA* wild-type patients. Additionally, identifying cancer types sensitive to combination treatments and optimizing combination dosages are key to the successful clinical application of combined PARPi and ATRi therapy in *BRCA1/2* wild-type cancer patients, necessitating further large-scale studies.

### 2.2 CHK inhibitors

Research indicates that DNA damage repair enables cells to respond to various stresses threatening genome stability. This response involves two main signaling pathways, ATM/Chk2 and ATR/CHK1, with the dysfunction of CHK1/2 closely linked to tumorigenesis (50, 51). CHK1 is a major effector downstream of ATR, becoming phosphorylated at Ser317 and Ser345 sites by the ATR kinase during replication stress-induced DNA damage (52, 53). While CHK2 acts as a serine/threonine kinase, it is a downstream phosphorylation substrate of ATM and is activated when DSB occur (54, 55). Further mechanistic studies showed that ATR-mediated phosphorylation and activation of CHK1 induced cell cycle arrest after DNA damage, which in turn led to the phosphorylation and degradation of cell division cycle factor 25 (CDC25), thereby inhibiting cell cycle progression to mitosis (M) and ultimately leading to cell cycle arrest to gain time for DNA repair. In contrast, when CHK1 is inhibited, CDC25 will be dephosphorylated, leading to the activation of C8/CDK complex. Although the presence of DNA damage will lead to programmed cell death, this complex will still drive cells to complete cell cycle progression. The primary functions of CHK1 not only involve regulating the cell cycle to prevent premature entry into the M phase but also stabilizing stalled replication forks and regulating HR (56, 57). Overall, CHK inhibitors (CHKi) play a crucial role in promoting cancer cells apoptosis through the aforementioned pathways.

In the process of homologous recombination repair after DNA damage, the binding of RAD51 to BRCA2 depends on the CHK1 phosphorylation of the C-terminal domain of BRCA2. RAD51 is responsible for regulating the invasion of ssDNA into the complementary parental strand to generate an extendable primer-dsDNA. CHK1-mediated phosphorylation of BRCA2 is important for the stability of BRCA2 and the efficient recruitment of RAD51 to sites of DNA damage, thereby facilitating effective DNA repair through homologous recombination (58, 59). CHK1 repairs excessive DNA damage in cancer cells, while inhibition of CHK1 leads to downregulation of DNA repair protein RAD51 and enhanced DNA damage (60). Therefore, CHK1i block the activation of HR by inhibiting the localization of RAD51 to nuclear lesions during DNA damage, thereby inhibiting the function of RAD51. The above studies elucidate the important role of CHK1i in blocking cell cycle progression, weakening replication forks, and inhibiting HR. DDR is coordinated by CHK1 and CHK2, acting independently to delay cell cycle progression and provide time for DNA repair. The ability of CHK1 to inhibit HR enhances the efficacy of PARPi on *BRCA* proficient tumors (61). Additionally, PARPi treatment upregulates p-ATR and p-CHK1, indicating the activation of the ATR-CHK1 protective pathway is critical in PARPi resistance. In fact, combination therapy with ATRi or CHK1i and PARPi synergistically reduces the survival and colony formation of BRCA wild-type cancer cells compared to monotherapy. Notably, PARPi treatment leads to cell cycle G2 arrest, while ATRi or CHK1i induces premature entry into mitosis, increasing chromosomal aberrations and cancer cell apoptosis (62). The combination of PARPi and CHK1i increases DSB and γH2AX expression. Studies have shown that ATM knockdown inhibits drug-induced CHK1 and ERK1/2 phosphorylation and enhances the cytotoxicity of PARPi and CHK1i on tumor cells (63) Prexasertib (LY2603618) is the first selective CHK1/2 inhibitor. Hye-Yon Cho found that the combination therapy of Prexasertib with Rucaparib exhibits significant anticancer effects in BRCA wild-type ovarian cancer cell lines, consistent with the study by Hyoung Kim (64). Based on these preclinical studies, the potential of CHK1i and PARPi combination therapy as a novel treatment for BRCA wild-type patients have been confirmed.

### 2.3 RAD51 inhibitor

Histone H2AX and RAD51 are key proteins involved in the DNA repair pathway (65, 66). Histone H2AX is phosphorylated into γH2AX, leading to the formation of other DNA repair proteins, such as RAD51, at the site of DSB (67, 68). Consequently, in preclinical and clinical samples, the formation of γH2AX and RAD51 foci are used as a biomarker for DSB (69). *RAD51* and its family play multiple roles in DSB repair, replication stress, and meiosis. Downregulation of RAD51 expression reduces the DNA damage repair capacity of tumor cells, thereby enhancing the efficacy of tumor gene toxic therapy (70). It has been reported that RAD51 is a key protein that mediates HR, and *RAD51* chromatin loading is the core step of HR (71). As a downstream effector molecule of the BRCA2 protein, RAD51 can be loaded onto ssDNA to promote the formation of RAD51-ssDNA nucleoprotein filaments and catalyze strand exchange reactions to initiate homology-directed repair (HDR) (72, 73). In fact, *RAD51* foci are known biomarkers for the HR repair pathway *in vitro*. When tumor cells are exposed to DNA-damaging agents, *RAD51* is recruited to the sites of DNA damage and forms distinct foci in a proficient HR repair environment (74). Targeting the specific interaction of RAD51-BRCA2 can mimic the effect of BRCA deficiency. Data suggest that in *BRCA1* wild-type TNBC, strategies targeting RAD51 can enhance the therapeutic efficacy of PARP inhibitors (75). Additionally, Scott et al. found that RAD51 inhibitor CAM833 enhances the damage effects of PARP inhibitors on *BRCA* wild-type cells by blocking the BRCA2-RAD51 protein interaction and preventing RAD51-mediated HR (76). In conclusion, RAD51 is the core protein of HR, and the combination of its inhibitor and PARPi is a promising treatment for *BRCA* wild-type tumors, which deserves further study.

### 2.4 WEE1 inhibitors

WEE1 is a type of tyrosine kinase, functioning as a key regulatory factor of the G2-M cell cycle checkpoint (77). The expression of WEE1 in cancer cells has dual biological roles. As a tumor suppressor, WEE1 can delay cell entry into mitosis by inhibiting CDK activity, thereby maintaining genomic stability. However, loss of WEE1 may lead to the accumulation of genetic abnormalities, promoting the development of pre-neoplastic lesions. As an oncogene, WEE1 promotes cancer cells to evade the effects of DNA damage and abnormal mitosis (78). WEE1 as an oncogene is highly expressed in various cancer types, including breast cancer. Cancer cells often exhibit defects in the G1-S checkpoint and heavily rely on the G2/M checkpoint to resist endogenous and exogenous DNA damage (79, 80). Activated WEE1 during DNA damage response maintains ATR and CHK1 phosphorylation to delay cell entry into mitosis (81). Similar to ATR, WEE1 is also involved in replication fork protection through direct interaction and negative regulation of DNA cleavage by endonuclease MUS81 (82). The MUS81 has a structure-specific activity for the Holliday junction formed during HR (83). Inhibition of WEE1 activates CDK1, leading to phosphorylation of BRCA2 and slowing down the progression of replication forks, thereby limiting HR (84–86). Since Adavosertib (AZD1775) usually regulates the G2/M checkpoint, the combination of Olaparib and AZD1775 significantly attenuates G2 arrest. AZD1775 reduces the expression of CtIP and RAD51 and disrupts HR repair (87). The increased susceptibility of cells to DNA damage induced by PARPi is due to the lack of CDK1/2-mediated phosphorylation-induced DNA repair defects and WEE1 inhibition(WEE1i).

Research has shown that combination therapy using PARPi and WEE1i synergistically induces cell death through replication stress and DNA damage (88). Cyclin E, as a key cell cycle regulatory factor, is a biomarker mediating replication stress in cancer. In tumor cells, it accumulates in the cytoplasm in a low molecular weight form. By co-administering Niraparib and WEE1i (Adavosertib) acting on Cyclin E, apoptosis in *BRCA* wild-type cells can be accelerated (89). Studies by Teo et al. have also demonstrated the synergistic effect of combining these two drugs in controlling tumor growth. The combination of Olaparib and Adavosertib triggers an increased anti-tumor immune response, including activation of the STING pathway. Combined use with STING (stimulator of interferon genes) agonists can further enhance persistent tumor regression in *BRCA1/2* wild-type TNBC mouse tumor models, significantly improving survival outcomes (90).

### 2.5 CDK inhibitors

The progression of the cell cycle largely depends on cyclin-dependent kinase (CDK), and the imbalance of CDK is associated with two key features of cancer cells, cell cycle dysregulation, and abnormal proliferation (91). The CDK-RB-E2F axis constitutes a central transcriptional mechanism that drives cell cycle progression, dictates the timing and fidelity of genome replication, and ensures the accurate transmission of genetic material in each cell division cycle (92). E2Fs are the main transcriptional regulatory factors of cell cycle-dependent gene expression, and they are highly active in almost all cancers, usually due to the inactivation of their main binding partner and key regulator RB (retinoblastoma), overexpression of CDK, or inactivation of CDK inhibitors (CDKi) (93, 94). Studies have confirmed that cyclin and its catalytic part control the transition between different stages of the cell cycle (95). Cyclin D1 is considered a key oncogenic driver in cancer (96), promoting the G1/S phase transition by binding and activating CDK4 and CDK6 (97). CDK4/6i effectively block cancer cell proliferation by inducing G1 cell cycle arrest (98). Furthermore, Cyclin D1 can inhibit Cyclin A-CDK2-dependent Ser329 phosphorylation and promote the binding of RAD51 to the C-terminal domain of BRCA2, while down-regulation of Cyclin D1 leads to low HR efficiency (99). Interfering with the HR repair pathway by blocking CDK1 can mimic *BRCA1* mutations and increase the sensitivity of TNBC cells to PARPi by 100-fold (100). Wild-type *BRCA* also participates in G1 cell cycle arrest. Aprelikova found that BRCA1 binds to hypophosphorylated RB and interacts with the E2F transcription factor to block transcription and inhibit cell proliferation (101). Research has shown that inhibiting CDK1, suppressed and transformed BRCA1 expression and phosphorylation transforms *BRCA* wild-type cancer cells into HR-deficient cells, making them more susceptible to synthetic lethality induced by PARPi (102, 103).

Additionally, the Johnson et al. discovered that the CDKi Dinaciclib reduces the expression of HR genes in *BRCA* wild-type TNBC cells and sensitizes these cells to Veliparib. Another study developed a dual PARP and CDK6 inhibitor named P4i, which is a new compound that links PARPi Olaparib and CDK6i Palbociclib through an o-phenylenedione moiety. This inhibitor significantly induces DNA damage and cell apoptosis, inhibiting the proliferation of TNBC cells through the signaling pathways involving PARP1 and CDK6 in *BRCA* wild-type cells (104). However, the interplay between the Cyclin/CDK pathway, HR, and *BRCA* wild-type remains complex, requiring further exploration and confirmation through future randomized clinical trials.

## 3 Inhibitors that indirectly suppress HR

### 3.1 VEGFR inhibitors

The vascular endothelial growth factor (VEGF or VEGF-A) was initially identified as a vascular permeability factor (VPF) and is one of the key molecules associated with angiogenesis (105). Patel pointed out that the angiogenic pathway, which develops new blood vessels from the existing vascular system, is a crucial step in tumor growth and metastasis (106). Studies have shown that hypoxia-induced by anti-angiogenic therapy inhibits HR repair by suppressing the expression of key factors such as *BRCA1* and *BRCA2*, leading to a deficiency in DDR and rendering *BRCA1/2* wild-type cancer cells sensitive to PARPi (107, 108). *BRCA* wild-type tumor cells are more sensitive to the VEGFR3 inhibitor Maz51. The addition of Maz51 can lead to *BRCA* gene down-regulation, inducing cell cycle arrest and leading to BRCAness, benefitting *BRCA* wild-type patients treated with PARPi (109). Furthermore, numerous studies have reported a synergistic effect of VEGFR inhibitors combined with PARPi in reducing the proliferation and invasion capabilities of tumor cells (110–112).

Early preclinical studies have shown that the anti-angiogenic agent Cediranib can inhibit the pro-survival and anti-apoptotic AKT signaling, significantly enhancing the inhibitory effects of ribonucleotide reductase inhibitor Triapine and Olaparib on *BRCA* wild-type epithelial ovarian cancer cells and extending the survival time of mice (113). Results from the clinical study NT02354131 also indicate that the combination of Bevacizumab and Niraparib is more effective than Niraparib monotherapy in treating *BRCA* wild-type ovarian cancer (114). Similarly, another phase III study showed that the combination of Bevacizumab and Olaparib than placebo plus bevacizumab significantly prolonged progression-free survival (PFS) in patients without *BRCA* gene mutation (28. 1 months vs 16. 6 months) (115). In addition, a phase II study also showed that the combination of Cediranib and Olaparib significantly improved the overall survival of patients with *BRCA1/2* wild-type ovarian cancer (37. 8 months vs 23. 0 months) (116). Subsequently, another phase III trial of J. F Liu showed that the remission rate of patients in the Olaparib/Cediranib combination group was significantly improved (117). Through preclinical and clinical studies, the efficacy of combining PARPi with VEGFR inhibitors have been widely recognized. Li et al. developed the first dual VEGFR/PARP inhibitor, which inhibits angiogenesis and invasion by negatively regulating the expression of VEGFR and PARP, thereby suppressing the growth and metastasis of *BRCA* wild-type breast cancer (118). These studies highlight the advantages of VEGFR/PARP dual inhibition in treating *BRCA* wild-type patients, with the potential to benefit more patients in the future. However, the exact mechanisms of combination therapy are not fully understood and may vary depending on the specific VEGFR inhibitors used, thus further research is needed to elucidate the precise mechanisms through which these combination therapies exert their anti-cancer effects.

### 3.2 EZH2 inhibitors

Epigenetic modifications are closely associated with tumorigenesis, mainly through regulating gene function and expression levels via DNA methylation and Histone modifications, thereby controlling cell differentiation (119). Enhancer of zeste homolog 2(EZH2) is the catalytic subunit of polycomb repressive complex 2(mainly composed of EZH2, embryonic ectoderm development (EED), suppressor of zeste 12 (SUZ12)), also a major regulator of cell cycle progression, autophagy, and apoptosis (120), and its overexpression can promote DNA damage repair and tumorigenesis (121). Therefore, any dysregulation of EZH2 might facilitate cancer development, while inhibiting its function and expression could make it an ideal target for epigenetic drug therapy. Numerous studies suggest that the combination of EZH2 inhibitors(EZH2i) and PARPi exhibits improved anticancer activity (122, 123). The typical mechanism involves tri-methylation of H3K27me3 by catalyzing EZH2, mediating transcriptional silencing to inhibit HR, thereby synergistically inducing synthetic lethality with PARPi (122, 124–128). Given the role of EZH2 as a transcriptional regulator, extensive research efforts have been devoted to identifying downstream targets or pathways driven by EZH2. In addition, mounting evidence indicates that EZH2 also plays a non-canonical role as a transcriptional activator, activating oncogenic pathways in a PRC2-independent manner, and directly modulating the activity of transcription factors and other proteins (129, 130). For instance, EZH2 transcriptionally upregulates IDH2 and promotes ovarian cancer growth by enhancing the tricarboxylic acid cycle activity to facilitate OXPHOS (131). However, the combination of EZH2i and PARPi can also elicit negative effects in the tumor microenvironment (TME). For example, the dual loss caused by PARP1 and EZH2 due to PRC2 deficiency exerts an oncogenic effect in *BRCA* wild-type breast cancer, primarily activating the NF-κB signaling pathway by forming a ternary complex with RelA and RelB (121), inducing the differentiation of tumor-promoting M2-type macrophages, disrupting the TME (132). Nevertheless, whether EZH2 inhibitors can fully suppress its biological functions to the extent of EZH2 loss remains inadequately studied.

GSK126 is one of the earliest discovered two selective EZH2i. It is more than 1000 times selective to EZH2 compared to other 20 methyltransferases, capable of reducing H3K27me3 levels and reactivating silenced *PRC2* target genes (133). By 2020, the latest generation of EZH2i Tazemetostat, also known as a competitive inhibitor of SAM, was approved by the FDA (134). Tazemetostat may have higher selectivity, better pharmacokinetic properties, better clinical efficacy and fewer side effects compared to GSK126, these advantages make it a more potential EZH2 inhibitor. In 2021, Wang et al. designed the first PARP and EZH2 dual inhibitor for treating *BRCA* wild-type TNBC, and its anti-proliferative activity was 15-80 times higher than that of Olaparib in *BRCA* wild-type cells (135), suggesting that the combination of EZH2i and PARPi holds promising prospects in the future.

### 3.3 HDAC inhibitors

As anti-tumor drugs, HDAC inhibitors(HDACi) can regulate the expression of HR-related genes, induce cell cycle arrest, apoptosis, and differentiation in tumor cells, leading to oxidative stress and DNA damage (136, 137). Preclinical studies have shown that Histone H3-Ser10 is a major target for ADP-ribosylation, and specific Histone acetylation marks have been found to block this activity. Among them, ADP-ribosylation induced by DNA damage is inhibited by jointly destroying post-translational modification (PTM) pathways such as acetylation-ADP-ribosylation (138). It has been reported that the HDACi SAHA induces acetylation of Histone H3 and induces degradation of the UHRF1 protein which is involved in maintenance DNA methylation and DNA damage repair. Combination therapy with Veliparib and SAHA can synergistically reduce the levels of BRCA1 by targeting the UHRF1/BRCA1 protein complex, and decreased UHRF1 levels can lead to BRCA1 protein degradation (139). New evidence indicates that dysregulation of HDAC function leads to downregulation of DNA repair genes such as *RAD51, BRCA1/2*, causing DNA repair defects and accumulation of DNA damage. Inhibition of HDAC can enhance the anti-tumor effect of PARPi in TNBC patients by blocking the DNA repair pathway (140).

Research has shown that the combination therapy of PARPi and HDACi significantly enhances the sensitivity of *BRCA* wild-type tumor cells to PARPi (141, 142). Synergistic effects of PARPi and HDACi have been observed in various cancer cells *in vitro* and *in vivo* studies. HDACi can inhibit DNA damage repair, downregulate HR and induce “BRCAness”, enhancing the biological activity of PARPi in TNBC regardless of *BRCA1* mutation status (143, 144). Researchers have found that the anti-tumor efficacy of HDACi is partially attributed to the downregulation of PARylation, inhibiting DNA repair proteins. This repair inhibition, combined with cancer cell-specific high levels of reactive oxygen species (ROS) and DNA replication stress, makes cancer cells highly sensitive to HDACi/PARPi combination therapy (145). As early as 2017, Yuan et al. constructed hydroxamic acid derivatives of Olaparib as dual inhibitors of PARP and HDAC, which significantly induced apoptosis in MDA-MB-231 cells (146). In conclusion, the combination therapy of PARPi and HDACi holds great promise as a cancer treatment, providing strong theoretical support for the treatment of *BRCA* wild-type patients, with more clinical trials to validate these research findings in the future.

### 3.4 BRD4 inhibitors

Bromodomain-containing protein 4 (BRD4) belongs to the bromodomain and extra-terminal domain (BET) family of proteins. Acting as a key factor in chromatin structure and transcription regulation, BRD4 plays a significant role in DNA damage repair and cell proliferation (147, 148). The survival of cancer cells is known to rely on aberrant transcription driven by super-enhancers (SEs), providing valuable targets for cancer treatment. Inhibiting BRD4 can disrupt the communication between SEs and promoters, effectively suppressing the transcription and expression of oncogenes, reducing cancer cell proliferation and viability. Specific inhibition of oncogenes leads to tumor cell death, making this mechanism the most recognized action of BET inhibitors(BETi) (149, 150). Further research has shown that inhibiting BRD4 can downregulate the transcription levels of 7 MTC(m6A methyltransferase complex) components, resulting in an overall decrease in m6A(N^6^-methyladenosine) modification. BETi significantly downregulate multiple genes in the HR pathway through the MTC-m6A mechanism, while also upregulating several pro-apoptotic genes. Studies using PDX models have revealed the synergistic effect of BETi/PARPi in targeting tumors through the BRD4-MTC-HR signaling axis (151). Recent studies indicate that regardless of *BRCA1/2* status, inhibiting BRD4 with BETi decreases the expression of the DNA repair factor CtIP, inducing HR defects and enhancing DNA damage induced by PARP inhibitors in cancer cells (152). These findings not only confirm the synergistic effects of BETi with DDR-targeted drugs but also demonstrate the potential of extending the efficacy of PARP inhibitors to non-*BRCA1/2* mutant cancers.

Yang et al. ‘s study demonstrates that inhibiting or depleting BET proteins affects the transcription of *BRCA1* and makes various tumor cells sensitive to PARP inhibitors (153). JQ1, as the first extensively studied BET family inhibitor, competitively binds to the acetylated lysine recognition motif or bromodomain (154). Preclinical studies indicate that the combination treatment of JQ1 with Olaparib can reduce the IC50 of Olaparib in OVCAR3 cells by approximately 50-fold, synergistically inhibiting the growth of *BRCA1/2* wild-type cells through inducing apoptosis (155). Further research reveals that simultaneous damage to the HR and BER pathways can induce significant death in *BRCA* wild-type TNBC cells. This implies that BET directly regulate HR-mediated DNA repair and induce the BRCAness phenotype in *BRCA1* wild-type TNBC cells (156). However, since many BRD4 inhibitors are pan-BET inhibitors rather than solely targeting BRD4, there is a risk of off-target effects. Therefore, the development of PARP/BRD4 dual inhibitors have become one of the future research directions. Wang et al. synthesized a highly selective PARP/BRD4 dual inhibitor, which displayed good synergistic anti-tumor efficacy in BRCA wild-type PDAC cells by blocking the cell cycle progression, inhibiting DNA damage repair, and promoting autophagy-related cell death (157). In 2022, another researcher designed a dual inhibitor (BP44) that can block G0/G1 transition and cell mitosis, reverse Olaparib-induced adaptive resistance, inhibit DNA damage repair, and promote DNA damage to induce death in *BRCA* wild-type TNBC cells (158). These findings collectively suggest that the combination of PARPi and agents specifically targeting BRD4 may present a novel strategy and direction for treating patients with *BRCA* wild-type cancers.

### 3.5 PI3K inhibitors

The dysregulation or mutation of the PI3K/AKT/mTOR pathway is one of the most common aberrant activation pathways in human malignancies, with increased PI3K signaling also considered as a hallmark of cancer (159, 160). Inhibiting the PI3K signaling pathway induces feedback upregulation of ERK, leading to increased activation of the ERK-related transcription factor ETS1 (161, 162). As ETS1 is a negative regulator of BRCA1/2 expression, the upregulation of ETS1 results in downregulation of BRCA and HR damage, making *BRCA* wild-type TNBC cells sensitive to PARP inhibitors (163–165). Studies have shown that treatment with Olaparib and Rucaparib leads to significant upregulation of the PI3K/mTOR pathway (p-mTOR, p-AKT, and pS6). Additionally, negative regulators of the PI3K pathway such as LKB1 and its targets AMPK and TSC are significantly downregulated. Following PARP depletion, phosphorylation of mTOR, AKT, and S6 increases while LKB1 signaling diminishes (166). Preclinical studies indicate that the combination of the PI3K inhibitor Buparlisib (NVP-BKM120) and Olaparib delays tumor proliferation in mouse models for over 70 days and in BRCA1-related tumor xenograft models for over 50 days, suggesting that combined PI3K and PARP inhibition could be an effective treatment for *BRCA1*-related tumors (167). BKM120 blocks PI3K by reducing HR ability, increasing ROS-mediated accumulation of γH2AX and DNA oxidative damage and inhibiting the expression of BRAC1/2 and RAD51/52 (168). BKM120 downregulates the expression of PARP1 and PARP2 to assist in PARP-mediated SSB repair blocking by Olaparib through the PI3K/Akt/NFκB/c-Myc signaling pathway and PI3K/Akt/FOXM1/Exo1 signaling pathway inhibiting HR. The combination of PI3K inhibitor BKM120 and Olaparib significantly reduces the proliferation of BRCA-proficient TNBC cells (169).

Based on successful preclinical studies, several clinical trials have demonstrated that the combination of PI3K/PARP inhibitors sensitizes *BRCA* wild-type TNBC, ovarian, and breast cancers to PARPi (170, 171). In 2020, researchers synthesized the first PARP/PI3K dual inhibitor, which can significantly inhibit the growth of *BRCA* wild-type cells by inhibiting the PI3K signaling pathway, down-regulating BRCA expression and inducing DNA damage and apoptosis (172). At present, an increasing number of dual inhibitors targeting *BRCA* wild-type cells have been developed, demonstrating superior anti-proliferative characteristics (173, 174).

### 3.6 Estrogen receptors

Estrogens regulate cell growth and development by acting on two different estrogen receptors ERα and ERβ. Among them, ERα can drive up to 70% of breast cancer, therefore targeting estrogen-positive receptors (ER+) is the standard approach for treating metastatic breast cancer (175, 176). Additionally, as a steroid hormone, estrogen plays a crucial role in maintaining sexual and overall health by regulating gene expression through interactions with transcription factor proteins in the cell nucleus rather than directly binding to DNA (177, 178). Since the *BRCA1* gene is a susceptibility gene for breast cancer, it can regulate the proliferation and differentiation of breast cells (179). During puberty and pregnancy, estrogen levels rise rapidly, leading to excessive mammary gland development and promoting the expression of *BRCA1* wild-type gene (180). Early studies indicate that by modulating the transcriptional activity of ERα to limit its stimulatory effect on proliferation of mammary epithelial cells, the occurrence of breast cancer can be effectively suppressed (181, 182). Furthermore, *BRCA1* wild-type can also reduce estrogen levels by inhibiting aromatase expression, thereby further decreasing ERα-mediated transcriptional activity (183).

As mentioned earlier, estrogen can inhibit cancer cells through various pathways. Recent research has shown that estrogen can suppress the expression of the *BRCA1* gene by stimulating the release of nitric oxide in ER+ breast cancer cells. This results in the accumulation of DSBs based on H2AX foci formation, reducing the HR repair mechanism in wild-type *BRCA* cancer cells, thereby enhancing the sensitivity of *BRCA* wild-type tumors to PARPi (184). On the other hand, as the most important and active hormone in estrogen, estradiol (E2) is closely related to the proliferation, differentiation and DNA damage of breast cells. Preclinical trials have shown that the combination of PARPi and E2 has a synergistic effect, with E2 enhancing PARPi-induced DNA damage and effectively inhibiting the recurrence of *BRCA1/2* wild-type tumors (185). Endocrine therapy for breast cancer was one of the earliest molecular targeted therapies used in cancer treatment, and besides causing endocrine-related symptoms, it does not lead to severe adverse events. The combination of PARPi and targeted estrogen therapy in breast cancer treatment has significant advantages and may become one of the important strategies in the field of personalized medicine in the future.

## 4 The combined application of PARP inhibitors and immune checkpoint inhibitors

PD-1 is a cell surface molecule that regulates adaptive immune responses. It transmits signals inhibiting T cell proliferation, cytokine production, and cytotoxic function by binding to its ligands, PD-L1 or PD-L2 (186, 187). High expression of PD-L1 serves as a prognostic biomarker for tumor progression and predicts the efficacy of immune checkpoint inhibitors (ICIs) in certain cancers (188). In recent years, multiple studies have also demonstrated an association between tumor immunity and HR, providing a theoretical basis for the combined use of PARPi and ICI (189–191). For instance, PD-L1 can promote DNA end resection to regulate HR in *BRCA1* wild-type tumor cells, enhancing HR repair capacity in tumor cells. Thus, the lack of PD-L1 can lead to increased DNA damage accumulation and improved tumor control of PARP inhibition in *BRCA1* wild-type tumors, while triggering synthetic lethality to PARP inhibitors *in vitro* (192).

On the other hand, PARPi can also indirectly activate dendritic cells by activating the cGAS-STING signaling pathway (193). PARPi increases the number of dendritic cells and enhances the antigen presentation mechanism by inducing the upregulation of two different signals, the co-stimulatory proteins CD80 and CD86, and MHC II (major histocompatibility complex class II), which mediates the presentation of antigens to T cells (194, 195). These two signals are also key components in the activation of naïve T cells, which after activation can control the expression of cell surface receptors such as CTLA-4 and PD-1 (196). PARPi-mediated DDR rapidly increases type I interferon expression through the cGAS-STING pathway and is a favorable factor in the treatment of ICI (197). DNA damage and cytoplasmic DNA-mediated cGAS- STING pathway contributes to the remodeling of the immune supportive environment. In addition, a series of studies have shown that PARPi-mediated DNA damage can enhance T cell recruitment and infiltration by activating the cGAS-STING pathway (194, 198, 199). For example, niraparib can activate STING-mediated type I interferon release and enhance T cell infiltration in tumors. In *BRCA* wild-type tumor models, inducing changes in the tumor microenvironment favored its combination with ICIs showing synergistic anti-tumor activity (200). Thus, combining PARPi to target these co-inhibitory pathways of ICIs in the context of cancer could be effective in achieving long-term anti-tumor effects. A mechanistic rationale for the use of PARPi as an immunomodulator to harness the therapeutic benefits of immunotherapy is provided.

The combination of ICI and PARPi has shown a synergistic effect in preclinical studies involving *BRCA* wild-type cancer, leading to the advancement of multiple clinical trials. A phase II clinical study of Olaparib and Durvalumab in the combination therapy for recurrent ovarian cancer has demonstrated that the combination of PARPi and anti-PD-L1 creates an immune stimulatory environment that can enhance durable anti-tumor immune response in the *BRCA* wild-type population toward immune checkpoint blockade (201). In the phase II trial (NCT03167619), the PFS of Olaparib in combination with Durvalumab was significantly longer compared to historical controls, with the subgroup of platinum-sensitive advanced TNBC patients with *BRCA* wild-type gaining sustained disease control (202). Another phase I and II clinical trials of Niraparib in combination with Pembrolizumab have shown an improved efficacy in *tBRCA* wild-type compared to monotherapy (ORR, 19%). Notably, among 8 patients with a response duration of over 6 months, 5 had platinum-refractory or platinum-resistant ovarian cancer and *tBRCA* wild-type tumors (203). Furthermore, the use of MEK inhibitors/PARPi combination or MEK inhibitors/PARPi/anti-PD-L1 triple therapy in *BRCA* wild-type tumor patients in NCT03695380 has demonstrated overall ORR and PFS superiority over single-agent Rucaparib (204, 205). The triple therapy combining ICI, PARPi, and Bevacizumab in the MEDIOLA trial (NCT02734004) has also shown promising activity, especially in *BRCA* wild-type tumor patients (206). The combination of ICI with PARPi may be a potential approach to enhance PARPi anti-tumor activity; however, these efficacy results still need to be confirmed in phase III clinical trials and compared to controls using PARPi alone.

## 5 The combined application of PARP inhibitors and chemotherapy

Chemotherapy, as a widely recognized cancer treatment method, typically uses cytotoxic drugs to treat various cancers (207). Chemotherapeutic agents are generally designed to kill tumor cells and prevent their proliferation, thereby inhibiting further growth and spread of the tumor. Chemotherapy can also cause DNA damage through various mechanisms, contributing adjunctively to enhancing the efficacy of other treatments, such as targeted therapies (208). For example, alkylating agents are the most common chemotherapy drugs, acting to chemically modify DNA at the level of base pairs. This DNA base damage may not immediately induce cytotoxic effects but rather induce cell cycle arrest by disrupting replication forks, leading to further cell damage like replication-associated DSB, mitotic catastrophe and cellular apoptosis. Considering the critical role of DSB repair pathways in cancer therapy resistance, inhibitors targeting different DSB repair pathways have been developed as potential sensitizers for conventional cancer treatments (209). Among them, PARPi play a pivotal role in blocking the DSB repair mechanism, which can maintain DSB damage and eventually lead to tumor cell death. Therefore, combination chemotherapy is more commonly used than single therapy.

Despite the strong theoretical basis for the combination therapy of PARPi and chemotherapy, it has not been proven to be as effective as other targeted HR inhibitors treatment strategies so far. One major issue is the narrow therapeutic window of drug treatment, as the synergistic effect of chemotherapy combined with PARPi is non-selective for tumor cells. Particularly, when PARPi is combined with platinum-based drugs, it enhances chemotherapy toxicity, including hematologic toxicity (210). A Phase III VELIA trial (NCT02470585) demonstrated no difference in ORR and PFS in the combination group compared to the control group in the *BRCA* wild-type population (211). Furthermore, clinical trials with paclitaxel regimens showed that weekly dosing improved PFS in *BRCA* wild-type patients compared to every three weeks dosing (carboplatin and paclitaxel with Veliparib) (18. 0 months vs 12. 9 months). However, while increasing the dosage to enhance efficacy, toxicity also increased accordingly (212). A Phase II clinical trial (NCT02595905) showed that adding Veliparib to cisplatin improved progression-free survival in *BRCA1/2* wild-type metastatic triple-negative breast cancer patients compared to cisplatin with placebo (18. 3% vs 4. 7%). Although the study showed that the Veliparib combination improved PFS, the exact conclusion on the efficacy of the combination therapy stage could not be drawn due to the potential impact of previous ICI treatment on Veliparib treatment effectiveness (213). The combination of chemotherapy and PARPi leads to additive toxicities such as bone marrow suppression, limiting patient treatment. Optimizing combination therapy regimens (including dosage and administration sequence) to reduce side effects while maintaining efficacy is a challenge that needs to be addressed when combining PARPi targeting HR repair with chemotherapy drugs.

## 6 The combined application of PARP inhibitors and radiotherapy

Radiotherapy (RT) is one of the cornerstones of cancer treatment, involving the use of high-energy ionizing radiation (IR) to kill cancer cells (214). The ideal treatment scenario involves selectively damaging only the tumor cells throughout the body while minimizing damage to healthy cells (215). However, the clustered DNA damage caused by IR does not always result in cancer cell death but rather triggers various complex DNA repair processes such as BER (216, 217). In such cases, PARPi are used as radiosensitizers to enhance the effects of radiation on tumors, improving anti-tumor responses with lower toxicity (218). The combination with ionizing radiation can enhance the radiosensitivity of various tumor cells, driving tumor cell death (219–224). The mechanism involves PARPi blocking the repair of radiation-induced damaged DNA through the BER pathway, increasing the likelihood of replication fork collapse to form persistent specific DSB and inhibiting HR and NHEJ repair pathways (225–228). Therefore, based on the ability of PARPi to amplify unrepaired DNA damage, the combination of PARPi and RT has become an effective treatment method (67, 229–231).

Different preclinical studies have shown that the combination of RT and PARPi is beneficial in the treatment of *BRCA* wild-type cells. Preclinical studies have demonstrated that the PARPi 3-aminobenzamide (3-AB) can enhance radiosensitivity in *BRCA* wild-type cell lines by blocking the repair of radiation-induced SSB (232). Additionally, the combination of Olaparib and PI-103 has enhanced radiation-induced cell death in *BRCA* wild-type cells (165). Another preclinical study showed that the combination of Olaparib, RT, and ATRi (AZD6738) significantly inhibited the growth of HR-proficient tumors (233). However, to date, only a limited number of preclinical studies have provided insights into the therapeutic potential of combining PARPi and RT for the treatment of *BRCA* wild-type cancer. Further investigation into the mechanism of action of this combination therapy is still needed in clinical trials. In conclusion, all these preclinical trials suggest that PARPi combined with radiotherapy is a promising strategy to enhance tumor DNA damage. By increasing DSB load through, PARPi combined with RT can make *BRCA* wild-type cancer cells radiosensitive and promote the death of these cells.

## 7 Conclusions

Although PARPi have achieved great success in treating patients with *BRCA1/2 mutated* cancers, the current application of PARPi still faces some challenges, especially in improving the efficacy of PARPi in *BRCA1/2 wild-type* cancer patients and overcoming acquired resistance to PARPi. Currently, early trials combining PARPi with targeted drugs such as ATRi, WEE1i, and VEGFRi have shown some progress. Additionally, research on the combination of PARPi with DNA-damaging agents such as cytotoxic chemotherapy and radiation therapy are progressing, but there are challenges of cumulative toxicity when PARPi is used in combination with DNA-damaging agents. Finally, some trials found that PARPi combined with immunotherapy can enhance anti-tumor immune responses and improve treatment outcomes. In summary, this review outlines the basic principles and ongoing clinical trials (**Table 1**) of PARPi in combination therapy with various agents, expecting that the indications for PARPi will be optimized and expanded in the coming years.

**Table 1:** Clinical trials in combination with PARPi for BRCA wild-type patients.

Trial identifier	Phase	Patient population	PARP inhibitor	Combination agent(s)	Reference
NCT03924245	I/II	Recurrent, Platinum-Refractory, Resistant Ovarian, Primary Peritoneal, Fallopian Tube Cancers	Olaparib	Entinostat	(234)
NCT03101280	I	Advanced Gynecologic Cancers and Triple-Negative Breast Cancer	Rucaparib	Atezolizumab	(235)
NCT02354131	I/II	Platinum-sensitive Epithelial Ovarian Cancer	Niraparib	Bevacizumab	(236)
NCT01968213	III	Relapsed High Grade Serous or Endometrioid Ovarian Cancer	Rucaparib	Placebo	(237)
NCT01116648	I/II	Recurrent Ovarian, Fallopian Tube, Peritoneal Cancer 、Recurrent TNBC	Olaparib	Cediranib Maleate	(238)
NCT02477644	III	Ovarian Cancer	Olaparib	platinum-taxane chemotherapy 、bevacizumab	(239)
NCT02446600	III	Platinum Sensitive Ovarian, Fallopian Tube, or Primary Peritoneal Cancer	Olaparib	Carboplatin、Cediranib 、 Gemcitabine	(240)
NCT04361370	II	BRCA Non-mutated Patients With Platinum-sensitive Recurrent Ovarian Cancer	Olaparib	Pembrolizumab、Bevacizumab	(241)
NCT01434316	I	Advanced Solid Tumors	Veliparib	Dinaciclib	(242)
NCT03462212	III	Advanced Ovarian, Primary Peritoneal and Fallopian Tube Cancer,	Rucaparib	Carboplatin、Paclitaxel Bevacizumab	(243)
NCT03740165	III	BRCA Non-mutated Advanced Epithelial Ovarian Cancer	Olaparib	Pembrolizumab、Carboplatin、Paclitaxel、Bevacizumab、Docetaxel	(244)
NCT03278717	III	Ovarian Cancer Patients	Olaparib	Cediranib	(245)
NCT03598270	III	Recurrent Ovarian Cancer	Paclitaxel	Carboplatin	(246)

## Author contributions

YX: Writing – original draft, Writing – review & editing. DX: Writing – original draft, Writing – review & editing. DL: Visualization, Writing – review & editing. MP: Visualization, Writing – review & editing. WP: Visualization, Writing – review & editing. HD: Writing – review & editing. XY: Writing – review & editing.

## References

[1] Sukhanova M Khodyreva S Lavrik O. Poly(Adp-ribose) polymerase 1 regulates activity of DNA polymerase beta in long patch base excision repair. Mutat Res. (2010) 685:80–9. doi: 10.1016/j.mrfmmm.2009.08.009 19703477

[2] Curtin NJ Szabo C. Poly(Adp-ribose) polymerase inhibition: Past, present and future. Nat Rev Drug Discovery. (2020) 19:711–36. doi: 10.1038/s41573-020-0076-6 32884152

[3] Paul S Sinha S Kundu CN. Targeting cancer stem cells in the tumor microenvironment: An emerging role of parp inhibitors. Pharmacol Res. (2022) 184:106425. doi: 10.1016/j.phrs.2022.106425 36075511

[4] Kawale AS Sung P. Mechanism and significance of chromosome damage repair by homologous recombination. Essays Biochem. (2020) 64:779–90. doi: 10.1042/EBC20190093 32756864

[5] Bell JC Kowalczykowski SC. Mechanics and single-molecule interrogation of DNA recombination. Annu Rev Biochem. (2016) 85:193–226. doi: 10.1146/annurev-biochem-060614-034352 27088880

[6] Farmer H McCabe N Lord CJ Tutt AN Johnson DA Richardson TB. Targeting the DNA repair defect in brca mutant cells as a therapeutic strategy. Nature. (2005) 434:917–21. doi: 10.1038/nature03445 15829967

[7] Bryant HE Schultz N Thomas HD Parker KM Flower D Lopez E. Specific killing of brca2-deficient tumours with inhibitors of poly(Adp-ribose) polymerase. Nature. (2005) 434:913–7. doi: 10.1038/nature03443 15829966

[8] Pilié PG Gay CM Byers LA O'Connor MJ Yap TA. Parp inhibitors: Extending benefit beyond brca-mutant cancers. Clin Cancer research: an Off J Am Assoc Cancer Res. (2019) 25:3759–71. doi: 10.1158/1078-0432.Ccr-18-0968 30760478

[9] Murai J Huang SY Das BB Renaud A Zhang Y Doroshow JH. Trapping of parp1 and parp2 by clinical parp inhibitors. Cancer Res. (2012) 72:5588–99. doi: 10.1158/0008-5472.CAN-12-2753 PMC3528345 23118055

[10] Pommier Y O'Connor MJ de Bono J. Laying a trap to kill cancer cells: Parp inhibitors and their mechanisms of action. Sci Trans Med. (2016) 8:362ps17. doi: 10.1126/scitranslmed.aaf9246 27797957

[11] Lord CJ Ashworth A. Parp inhibitors: Synthetic lethality in the clinic. Sci (New York NY). (2017) 355:1152–8. doi: 10.1126/science.aam7344 PMC6175050 28302823

[12] Sullivan-Reed K Bolton-Gillespie E Dasgupta Y Langer S Siciliano M Nieborowska-Skorska M. Simultaneous targeting of parp1 and rad52 triggers dual synthetic lethality in brca-deficient tumor cells. Cell Rep. (2018) 23:3127–36. doi: 10.1016/j.celrep.2018.05.034 PMC6082171 29898385

[13] Arcieri M Tius V Andreetta C Restaino S Biasioli A Poletto E. How brca and homologous recombination deficiency change therapeutic strategies in ovarian cancer: A review of literature. Front Oncol. (2024) 14:1335196. doi: 10.3389/fonc.2024.1335196 38525421 PMC10957789

[14] Pietragalla A Arcieri M Marchetti C Scambia G Fagotti A. Ovarian cancer predisposition beyond brca1 and brca2 genes. Int J gynecological cancer: Off J Int Gynecological Cancer Soc. (2020) 30:1803–10. doi: 10.1136/ijgc-2020-001556 32895312

[15] Friedlander M Matulonis U Gourley C du Bois A Vergote I Rustin G. Long-term efficacy, tolerability and overall survival in patients with platinum-sensitive, recurrent high-grade serous ovarian cancer treated with maintenance olaparib capsules following response to chemotherapy. Br J Cancer. (2018) 119:1075–85. doi: 10.1038/s41416-018-0271-y PMC6219499 30353045

[16] Zhang J Gao Y Zhang Z Zhao J Jia W Xia C. Multi-therapies based on parp inhibition: Potential therapeutic approaches for cancer treatment. J medicinal Chem. (2022) 65:16099–127. doi: 10.1021/acs.jmedchem.2c01352 36512711

[17] Ashworth A. A synthetic lethal therapeutic approach: Poly(Adp) ribose polymerase inhibitors for the treatment of cancers deficient in DNA double-strand break repair. J Clin oncology: Off J Am Soc Clin Oncol. (2008) 26:3785–90. doi: 10.1200/JCO.2008.16.0812 18591545

[18] Lee JH Paull TT. Direct activation of the atm protein kinase by the mre11/rad50/nbs1 complex. Sci (New York NY). (2004) 304:93–6. doi: 10.1126/science.1091496 15064416

[19] Lee JH Paull TT. Atm activation by DNA double-strand breaks through the mre11-rad50-nbs1 complex. Sci (New York NY). (2005) 308:551–4. doi: 10.1126/science.1108297 15790808

[20] Wang XS Menolfi D Wu-Baer F Fangazio M Meyer SN Shao Z. DNA damage-induced phosphorylation of ctip at a conserved atm/atr site T855 promotes lymphomagenesis in mice. Proc Natl Acad Sci United States America. (2021) 118:e2105440118. doi: 10.1073/pnas.2105440118 PMC8463888 34521752

[21] Gao M Guo G Huang J Kloeber JA Zhao F Deng M. Usp52 regulates DNA end resection and chemosensitivity through removing inhibitory ubiquitination from ctip. Nat Commun. (2020) 11:5362. doi: 10.1038/s41467-020-19202-0 33097710 PMC7584643

[22] Luo SC Yeh MC Lien YH Yeh HY Siao HL Tu IP. A rad51-adp double filament structure unveils the mechanism of filament dynamics in homologous recombination. Nat Commun. (2023) 14:4993. doi: 10.1038/s41467-023-40672-5 37591853 PMC10435448

[23] McCabe N Turner NC Lord CJ Kluzek K Bialkowska A Swift S. Deficiency in the repair of DNA damage by homologous recombination and sensitivity to poly(Adp-ribose) polymerase inhibition. Cancer Res. (2006) 66:8109–15. doi: 10.1158/0008-5472.CAN-06-0140 16912188

[24] Zhang P Li R Xiao H Liu W Zeng X Xie G. Brd4 inhibitor azd5153 suppresses the proliferation of colorectal cancer cells and sensitizes the anticancer effect of parp inhibitor. Int J Biol Sci. (2019) 15:1942–54. doi: 10.7150/ijbs.34162 PMC6743290 31523195

[25] Karakashev S Fukumoto T Zhao B Lin J Wu S Fatkhutdinov N. Ezh2 inhibition sensitizes carm1-high, homologous recombination proficient ovarian cancers to parp inhibition. Cancer Cell. (2020) 37:157–67.e6. doi: 10.1016/j.ccell.2019.12.015 32004442 PMC7155421

[26] Min A Im SA Kim DK Song SH Kim HJ Lee KH. Histone deacetylase inhibitor, suberoylanilide hydroxamic acid (Saha), enhances anti-tumor effects of the poly (Adp-ribose) polymerase (Parp) inhibitor olaparib in triple-negative breast cancer cells. Breast Cancer research: BCR. (2015) 17:33. doi: 10.1186/s13058-015-0534-y 25888415 PMC4425881

[27] Cimprich KA Cortez D. Atr: an essential regulator of genome integrity. Nat Rev Mol Cell Biol. (2008) 9:616–27. doi: 10.1038/nrm2450 PMC2663384 18594563

[28] Jackson SP Bartek J. The DNA-damage response in human biology and disease. Nature. (2009) 461:1071–8. doi: 10.1038/nature08467 PMC2906700 19847258

[29] Yano K Shiotani B. Emerging strategies for cancer therapy by atr inhibitors. Cancer Sci. (2023) 114:2709–21. doi: 10.1111/cas.15845 PMC10323102 37189251

[30] Saldivar JC Hamperl S Bocek MJ Chung M Bass TE Cisneros-Soberanis F. An intrinsic S/G(2) checkpoint enforced by atr. Sci (New York NY). (2018) 361:806–10. doi: 10.1126/science.aap9346 PMC6365305 30139873

[31] Sørensen CS Syljuåsen RG. Safeguarding genome integrity: The checkpoint kinases atr, chk1 and wee1 restrain cdk activity during normal DNA replication. Nucleic Acids Res. (2012) 40:477–86. doi: 10.1093/nar/gkr697 PMC3258124 21937510

[32] Saldivar JC Cortez D Cimprich KA. The essential kinase atr: Ensuring faithful duplication of a challenging genome. Nat Rev Mol Cell Biol. (2017) 18:622–36. doi: 10.1038/nrm.2017.67 PMC5796526 28811666

[33] Leung W Simoneau A Saxena S Jackson J Patel PS Limbu M. Atr protects ongoing and newly assembled DNA replication forks through distinct mechanisms. Cell Rep. (2023) 42:112792. doi: 10.1016/j.celrep.2023.112792 37454295 PMC10529362

[34] Tibbetts RS Cortez D Brumbaugh KM Scully R Livingston D Elledge SJ. Functional interactions between brca1 and the checkpoint kinase atr during genotoxic stress. Genes Dev. (2000) 14:2989–3002. doi: 10.1101/gad.851000 11114888 PMC317107

[35] Wang A Schneider-Broussard R Kumar AP MacLeod MC Johnson DG. Regulation of brca1 expression by the rb-E2f pathway. J Biol Chem. (2000) 275:4532–6. doi: 10.1074/jbc.275.6.4532 10660629

[36] Bertoli C Klier S McGowan C Wittenberg C de Bruin RA. Chk1 inhibits E2f6 repressor function in response to replication stress to maintain cell-cycle transcription. Curr biology: CB. (2013) 23:1629–37. doi: 10.1016/j.cub.2013.06.063 PMC3977652 23954429

[37] Kim D Liu Y Oberly S Freire R Smolka MB. Atr-mediated proteome remodeling is a major determinant of homologous recombination capacity in cancer cells. Nucleic Acids Res. (2018) 46:8311–25. doi: 10.1093/nar/gky625 PMC6144784 30010936

[38] Buisson R Niraj J Rodrigue A Ho CK Kreuzer J Foo TK. Coupling of homologous recombination and the checkpoint by atr. Mol Cell. (2017) 65:336–46. doi: 10.1016/j.molcel.2016.12.007 PMC5496772 28089683

[39] Egger T Bordignon B Coquelle A. A clinically relevant heterozygous atr mutation sensitizes colorectal cancer cells to replication stress. Sci Rep. (2022) 12:5422. doi: 10.1038/s41598-022-09308-4 35361811 PMC8971416

[40] Chen H Pan T Zheng X Huang Y Wu C Yang T. The atr-wee1 kinase module promotes suppressor of gamma response 1 translation to activate replication stress responses. Plant Cell. (2023) 35:3021–34. doi: 10.1093/plcell/koad126 PMC10396359 37159556

[41] Randi GS Grete H Sissel H Åslaug H. Targeting lung cancer through inhibition of checkpoint kinases. Front Genet. (2015) 6:70. doi: 10.3389/fgene.2015.00070 25774168 PMC4343027

[42] Gralewska P Gajek A Marczak A Mikuła M Ostrowski J Śliwińska A. Parp inhibition increases the reliance on atr/chk1 checkpoint signaling leading to synthetic lethality-an alternative treatment strategy for epithelial ovarian cancer cells independent from hr effectiveness. Int J Mol Sci. (2020) 21:9715. doi: 10.3390/ijms21249715 PMC7766831 33352723

[43] Qiu Z Oleinick NL Zhang J. Atr/chk1 inhibitors and cancer therapy. Radiotherapy oncology: J Eur Soc Ther Radiol Oncol. (2018) 126:450–64. doi: 10.1016/j.radonc.2017.09.043 PMC5856582 29054375

[44] Mohni KN Thompson PS Luzwick JW Glick GG Pendleton CS Lehmann BD. A synthetic lethal screen identifies DNA repair pathways that sensitize cancer cells to combined atr inhibition and cisplatin treatments. PLoS One. (2015) 10:e0125482. doi: 10.1371/journal.pone.0125482 25965342 PMC4428765

[45] Zimmermann M Bernier C Kaiser B Fournier S Li L Desjardins J. Guiding atr and parp inhibitor combinationswith chemogenomic screens. Cell Rep. (2022) 40:111081. doi: 10.1016/j.celrep.2022.111081 35830811

[46] Mah LJ El-Osta A Karagiannis TC. Gammah2ax: A sensitive molecular marker of DNA damage and repair. Leukemia. (2010) 24:679–86. doi: 10.1038/leu.2010.6 20130602

[47] Nam AR Jin MH Park JE Bang JH Oh DY Bang YJ. Therapeutic targeting of the DNA damage response using an atr inhibitor in biliary tract cancer. Cancer Res Treat. (2019) 51:1167–79. doi: 10.4143/crt.2018.526 PMC6639230 30514066

[48] Wilson Z Odedra R Wallez Y Wijnhoven PWG Hughes AM Gerrard J. Atr inhibitor azd6738 (Ceralasertib) exerts antitumor activity as a monotherapy and in combination with chemotherapy and the parp inhibitor olaparib. Cancer Res. (2022) 82:1140–52. doi: 10.1158/0008-5472.CAN-21-2997 PMC9359726 35078817

[49] Kim H Xu H George E Hallberg D Kumar S Jagannathan V. Combining parp with atr inhibition overcomes parp inhibitor and platinum resistance in ovarian cancer models. Nat Commun. (2020) 11:3726. doi: 10.1038/s41467-020-17127-2 32709856 PMC7381609

[50] Yin Y Shen Q Zhang P Tao R Chang W Li R. Chk1 inhibition potentiates the therapeutic efficacy of parp inhibitor bmn673 in gastric cancer. Am J Cancer Res. (2017) 7:473–83. PMC5385637 28401005

[51] Kim MA Kim HJ Brown AL Lee MY Bae YS Park JI. Identification of novel substrates for human checkpoint kinase chk1 and chk2 through genome-wide screening using a consensus chk phosphorylation motif. Exp Mol Med. (2007) 39:205–12. doi: 10.1038/emm.2007.23 17464182

[52] Kim PR Koon YL Lee RTC Azizan F Koh DHZ Chiam KH. Phosphatase popx2 interferes with cell cycle by interacting with chk1. Cell Cycle (Georgetown Tex). (2020) 19:405–18. doi: 10.1080/15384101.2020.1711577 PMC7100883 31944151

[53] Guillou E Ibarra A Coulon V Casado-Vela J Rico D Casal I. Cohesin organizes chromatin loops at DNA replication factories. Genes Dev. (2010) 24:2812–22. doi: 10.1101/gad.608210 PMC3003199 21159821

[54] Liu F Pan R Ding H Gu L Yang Y Li C. Ubqln4 is an atm substrate that stabilizes the anti-apoptotic proteins bcl2a1 and bcl2l10 in mesothelioma. Mol Oncol. (2021) 15:3738–52. doi: 10.1002/1878-0261.13058 PMC8637560 34245648

[55] Gąsior-Perczak D Kowalik A Walczyk A Siołek M Gruszczyński K Pałyga I. Coexisting germline chek2 and somatic braf(V600e) mutations in papillary thyroid cancer and their association with clinicopathological features and disease course. Cancers. (2019) 11:1744. doi: 10.3390/cancers11111744 PMC6896084 31703344

[56] Scorah J McGowan CH. Claspin and chk1 regulate replication fork stability by different mechanisms. Cell Cycle (Georgetown Tex). (2009) 8:1036–43. doi: 10.4161/cc.8.7.8040 PMC2668962 19270516

[57] Petermann E Woodcock M Helleday T. Chk1 promotes replication fork progression by controlling replication initiation. Proc Natl Acad Sci United States America. (2010) 107:16090–5. doi: 10.1073/pnas.1005031107 PMC2941317 20805465

[58] Bahassi EM Ovesen JL Riesenberg AL Bernstein WZ Hasty PE Stambrook PJ. The checkpoint kinases chk1 and chk2 regulate the functional associations between hbrca2 and rad51 in response to DNA damage. Oncogene. (2008) 27:3977–85. doi: 10.1038/onc.2008.17 18317453

[59] Sørensen CS Hansen LT Dziegielewski J Syljuåsen RG Lundin C Bartek J. The cell-cycle checkpoint kinase chk1 is required for mammalian homologous recombination repair. Nat Cell Biol. (2005) 7:195–201. doi: 10.1038/ncb1212 15665856

[60] Bourgeois A Bonnet S Breuils-Bonnet S Habbout K Paradis R Tremblay E. Inhibition of chk 1 (Checkpoint kinase 1) elicits therapeutic effects in pulmonary arterial hypertension. Arteriosclerosis thrombosis Vasc Biol. (2019) 39:1667–81. doi: 10.1161/ATVBAHA.119.312537 PMC6727643 31092016

[61] Neizer-Ashun F Bhattacharya R. Reality chek: understanding the biology and clinical potential of chk1. Cancer Lett. (2021) 497:202–11. doi: 10.1016/j.canlet.2020.09.016 32991949

[62] Kim H George E Ragland R Rafail S Zhang R Krepler C. Targeting the atr/chk1 axis with parp inhibition results in tumor regression in brca-mutant ovarian cancer models. Clin Cancer research: an Off J Am Assoc Cancer Res. (2017) 23:3097–108. doi: 10.1158/1078-0432.CCR-16-2273 PMC5474193 27993965

[63] Booth L Cruickshanks N Ridder T Dai Y Grant S Dent P. Parp and chk inhibitors interact to cause DNA damage and cell death in mammary carcinoma cells. Cancer Biol Ther. (2013) 14:458–65. doi: 10.4161/cbt.24424 PMC3672190 23917378

[64] Cho HY Kim YB Park WH No JH. Enhanced efficacy of combined therapy with checkpoint kinase 1 inhibitor and rucaparib via regulation of rad51 expression in brca wild-type epithelial ovarian cancer cells. Cancer Res Treat. (2021) 53:819–28. doi: 10.4143/crt.2020.1013 PMC8291182 33332934

[65] Gudmundsdottir K Ashworth A. The roles of brca1 and brca2 and associated proteins in the maintenance of genomic stability. Oncogene. (2006) 25:5864–74. doi: 10.1038/sj.onc.1209874 16998501

[66] Bonner WM Redon CE Dickey JS Nakamura AJ Sedelnikova OA Solier S. Gammah2ax and cancer. Nat Rev Cancer. (2008) 8:957–67. doi: 10.1038/nrc2523 PMC3094856 19005492

[67] Fong PC Boss DS Yap TA Tutt A Wu P Mergui-Roelvink M. Inhibition of poly(Adp-ribose) polymerase in tumors from brca mutation carriers. New Engl J Med. (2009) 361:123–34. doi: 10.1056/NEJMoa0900212 19553641

[68] Bañuelos CA Banáth JP Kim JY Aquino-Parsons C Olive PL. Gammah2ax expression in tumors exposed to cisplatin and fractionated irradiation. Clin Cancer research: an Off J Am Assoc Cancer Res. (2009) 15:3344–53. doi: 10.1158/1078-0432.Ccr-08-3114 19401347

[69] Rogakou EP Pilch DR Orr AH Ivanova VS Bonner WM. DNA double-stranded breaks induce histone H2ax phosphorylation on serine 139. J Biol Chem. (1998) 273:5858–68. doi: 10.1074/jbc.273.10.5858 9488723

[70] Bonilla B Hengel SR Grundy MK Bernstein KA. Rad51 gene family structure and function. Annu Rev Genet. (2020) 54:25–46. doi: 10.1146/annurev-genet-021920-092410 32663049 PMC7703940

[71] Hariharasudhan G Jeong SY Kim MJ Jung SM Seo G Moon JR. Topors-mediated rad51 sumoylation facilitates homologous recombination repair. Nucleic Acids Res. (2022) 50:1501–16. doi: 10.1093/nar/gkac009 PMC8860612 35061896

[72] Kowalczykowski SC. An overview of the molecular mechanisms of recombinational DNA repair. Cold Spring Harbor Perspect Biol. (2015) 7:a016410. doi: 10.1101/cshperspect.a016410 PMC4632670 26525148

[73] Wang Z Jia R Wang L Yang Q Hu X Fu Q. The emerging roles of rad51 in cancer and its potential as a therapeutic target. Front Oncol. (2022) 12:935593. doi: 10.3389/fonc.2022.935593 35875146 PMC9300834

[74] Cruz C Castroviejo-Bermejo M Gutiérrez-Enríquez S Llop-Guevara A Ibrahim YH Gris-Oliver A. Rad51 foci as a functional biomarker of homologous recombination repair and parp inhibitor resistance in germline brca-mutated breast cancer. Ann oncology: Off J Eur Soc Med Oncol. (2018) 29:1203–10. doi: 10.1093/annonc/mdy099 PMC5961353 29635390

[75] Liu Y Burness ML Martin-Trevino R Guy J Bai S Harouaka R. Rad51 mediates resistance of cancer stem cells to parp inhibition in triple-negative breast cancer. Clin Cancer research: an Off J Am Assoc Cancer Res. (2017) 23:514–22. doi: 10.1158/1078-0432.CCR-15-1348 28034904

[76] Scott DE Francis-Newton NJ Marsh ME Coyne AG Fischer G Moschetti T. A small-molecule inhibitor of the brca2-rad51 interaction modulates rad51 assembly and potentiates DNA damage-induced cell death. Cell Chem Biol. (2021) 28:835–47.e5. doi: 10.1016/j.chembiol.2021.02.006 33662256 PMC8219027

[77] Magnussen GI Holm R Emilsen E Rosnes AK Slipicevic A Flørenes VA. High expression of wee1 is associated with poor disease-free survival in Malignant melanoma: potential for targeted therapy. PLoS One. (2012) 7:e38254. doi: 10.1371/journal.pone.0038254 22719872 PMC3373567

[78] Ghelli Luserna Di Rorà A Beeharry N Imbrogno E Ferrari A Robustelli V Righi S. Targeting wee1 to enhance conventional therapies for acute lymphoblastic leukemia. J Hematol Oncol. (2018) 11:99. doi: 10.1186/s13045-018-0641-1 30068368 PMC6090987

[79] Matheson CJ Backos DS Reigan P. Targeting wee1 kinase in cancer. Trends Pharmacol Sci. (2016) 37:872–81. doi: 10.1016/j.tips.2016.06.006 27427153

[80] Bukhari AB Chan GK Gamper AM. Targeting the DNA damage response for cancer therapy by inhibiting the kinase wee1. Front Oncol. (2022) 12:828684. doi: 10.3389/fonc.2022.828684 35251998 PMC8891215

[81] Saini P Li Y Dobbelstein M. Wee1 is required to sustain atr/chk1 signaling upon replicative stress. Oncotarget. (2015) 6:13072–87. doi: 10.18632/oncotarget.v6i15 PMC4537000 25965828

[82] Domínguez-Kelly R Martín Y Koundrioukoff S Tanenbaum ME Smits VA Medema RH. Wee1 controls genomic stability during replication by regulating the mus81-eme1 endonuclease. J Cell Biol. (2011) 194:567–79. doi: 10.1083/jcb.201101047 PMC3160579 21859861

[83] Taylor ER McGowan CH. Cleavage mechanism of human mus81-eme1 acting on holliday-junction structures. Proc Natl Acad Sci United States America. (2008) 105:3757–62. doi: 10.1073/pnas.0710291105 PMC2268786 18310322

[84] Hirai H Arai T Okada M Nishibata T Kobayashi M Sakai N. Mk-1775, a small molecule wee1 inhibitor, enhances anti-tumor efficacy of various DNA-damaging agents, including 5-fluorouracil. Cancer Biol Ther. (2010) 9:514–22. doi: 10.4161/cbt.9.7.11115 20107315

[85] Krajewska M Heijink AM Bisselink YJ Seinstra RI Silljé HH de Vries EG. Forced activation of cdk1 via wee1 inhibition impairs homologous recombination. Oncogene. (2013) 32:3001–8. doi: 10.1038/onc.2012.296 22797065

[86] Kausar T Schreiber JS Karnak D Parsels LA Parsels JD Davis MA. Sensitization of pancreatic cancers to gemcitabine chemoradiation by wee1 kinase inhibition depends on homologous recombination repair. Neoplasia (New York NY). (2015) 17:757–66. doi: 10.1016/j.neo.2015.09.006 PMC4656803 26585231

[87] Seo HR Nam AR Bang JH Oh KS Kim JM Yoon J. Inhibition of wee1 potentiates sensitivity to parp inhibitor in biliary tract cancer. Cancer Res Treat. (2022) 54:541–53. doi: 10.4143/crt.2021.473 PMC9016294 34352995

[88] Fang Y McGrail DJ Sun C Labrie M Chen X Zhang D. Sequential therapy with parp and wee1 inhibitors minimizes toxicity while maintaining efficacy. Cancer Cell. (2019) 35:851–67.e7. doi: 10.1016/j.ccell.2019.05.001 31185210 PMC6642675

[89] Chen X Yang D Carey JPW Karakas C Albarracin C Sahin AA. Targeting replicative stress and DNA repair by combining parp and wee1 kinase inhibitors is synergistic in triple negative breast cancers with cyclin E or brca1 alteration. Cancers. (2021) 13:1656. doi: 10.3390/cancers13071656 PMC8036262 33916118

[90] Teo ZL O'Connor MJ Versaci S Clarke KA Brown ER Percy LW. Combined parp and wee1 inhibition triggers anti-tumor immune response in brca1/2 wildtype triple-negative breast cancer. NPJ Breast Cancer. (2023) 9:68. doi: 10.1038/s41523-023-00568-5 37582853 PMC10427618

[91] Mughal MJ Bhadresha K Kwok HF. Cdk inhibitors from past to present: A new wave of cancer therapy. Semin Cancer Biol. (2023) 88:106–22. doi: 10.1016/j.semcancer.2022.12.006 36565895

[92] Kent LN Leone G. The broken cycle: E2f dysfunction in cancer. Nat Rev Cancer. (2019) 19:326–38. doi: 10.1038/s41568-019-0143-7 31053804

[93] Azechi H Nishida N Fukuda Y Nishimura T Minata M Katsuma H. Disruption of the P16/cyclin D1/retinoblastoma protein pathway in the majority of human hepatocellular carcinomas. Oncology. (2001) 60:346–54. doi: 10.1159/000058531 11408803

[94] Di Fiore R D'Anneo A Tesoriere G Vento R. Rb1 in cancer: different mechanisms of rb1 inactivation and alterations of prb pathway in tumorigenesis. J Cell Physiol. (2013) 228:1676–87. doi: 10.1002/jcp.24329 23359405

[95] Bury M Le Calvé B Ferbeyre G Blank V Lessard F. New insights into cdk regulators: novel opportunities for cancer therapy. Trends Cell Biol. (2021) 31:331–44. doi: 10.1016/j.tcb.2021.01.010 33676803

[96] Jun SY Kim J Yoon N Maeng LS Byun JH. Prognostic potential of cyclin D1 expression in colorectal cancer. J Clin Med. (2023) 12:572. doi: 10.3390/jcm12020572 PMC9867305 36675501

[97] Fu M Wang C Li Z Sakamaki T Pestell RG. Minireview: cyclin D1: Normal and abnormal functions. Endocrinology. (2004) 145:5439–47. doi: 10.1210/en.2004-0959 15331580

[98] Ribnikar D Volovat SR Cardoso F. Targeting cdk4/6 pathways and beyond in breast cancer. Breast (Edinburgh Scotland). (2019) 43:8–17. doi: 10.1016/j.breast.2018.10.001 30359883

[99] Chalermrujinanant C Michowski W Sittithumcharee G Esashi F Jirawatnotai S. Cyclin D1 promotes brca2-rad51 interaction by restricting cyclin a/B-dependent brca2 phosphorylation. Oncogene. (2016) 35:2815–23. doi: 10.1038/onc.2015.354 PMC6538526 26387543

[100] Kciuk M Gielecińska A Mujwar S Mojzych M Kontek R. Cyclin-dependent kinase synthetic lethality partners in DNA damage response. Int J Mol Sci. (2022) 23:3555. doi: 10.3390/ijms23073555 PMC8998982 35408915

[101] Aprelikova ON Fang BS Meissner EG Cotter S Campbell M Kuthiala A. Brca1-associated growth arrest is rb-dependent. Proc Natl Acad Sci United States America. (1999) 96:11866–71. doi: 10.1073/pnas.96.21.11866 PMC18378 10518542

[102] Johnson N Li YC Walton ZE Cheng KA Li D Rodig SJ. Compromised cdk1 activity sensitizes brca-proficient cancers to parp inhibition. Nat Med. (2011) 17:875–82. doi: 10.1038/nm.2377 PMC3272302 21706030

[103] Johnson SF Cruz C Greifenberg AK Dust S Stover DG Chi D. Cdk12 inhibition reverses *de novo* and acquired parp inhibitor resistance in brca wild-type and mutated models of triple-negative breast cancer. Cell Rep. (2016) 17:2367–81. doi: 10.1016/j.celrep.2016.10.077 PMC5176643 27880910

[104] Wang C Luo H Chen X Zhang Y Lu D Liu X. Discovery of dual parp and cdk6 inhibitors for triple-negative breast cancer with wild-type brca. Bioorganic Chem. (2023) 139:106683. doi: 10.1016/j.bioorg.2023.106683 37379778

[105] Saikia Q Reeve H Alzahrani A Critchley WR Zeqiraj E Divan A. Vegfr endocytosis: implications for angiogenesis. Prog Mol Biol Trans Sci. (2023) 194:109–39. doi: 10.1016/bs.pmbts.2022.06.021 36631189

[106] Patel SA Nilsson MB Le X Cascone T Jain RK Heymach JV. Molecular mechanisms and future implications of vegf/vegfr in cancer therapy. Clin Cancer research: an Off J Am Assoc Cancer Res. (2023) 29:30–9. doi: 10.1158/1078-0432.CCR-22-1366 PMC10274152 35969170

[107] An D Banerjee S Lee JM. Recent advancements of antiangiogenic combination therapies in ovarian cancer. Cancer Treat Rev. (2021) 98:102224. doi: 10.1016/j.ctrv.2021.102224 34051628 PMC8217312

[108] Chatterjee S Sinha S Molla S Hembram KC Kundu CN. Parp inhibitor veliparib (Abt-888) enhances the anti-angiogenic potentiality of curcumin through deregulation of nectin-4 in oral cancer: role of nitric oxide (No). Cell signalling. (2021) 80:109902. doi: 10.1016/j.cellsig.2020.109902 33373686

[109] Lim JJ Yang K Taylor-Harding B Wiedemeyer WR Buckanovich RJ. Vegfr3 inhibition chemosensitizes ovarian cancer stemlike cells through down-regulation of brca1 and brca2. Neoplasia (New York NY). (2014) 16:343–53.e1-2. doi: 10.1016/j.neo.2014.04.003 PMC4094836 24862760

[110] Suminokura J Miyamoto M Yoshikawa T Kouta H Kikuchi Y Hada T. Potential efficacy of weekly low-dose administration of bevacizumab as a combination therapy for platinum-resistant ovarian carcinoma: A retrospective analysis. BMC Cancer. (2022) 22:176. doi: 10.1186/s12885-022-09271-3 35172766 PMC8849038

[111] McCann KE. Novel poly-adp-ribose polymerase inhibitor combination strategies in ovarian cancer. Curr Opin obstetrics gynecology. (2018) 30:7–16. doi: 10.1097/GCO.0000000000000428 29251678

[112] Gourd E. Olaparib plus bevacizumab improves progression-free survival in ovarian cancer. Lancet Oncol. (2020) 21:e71. doi: 10.1016/S1470-2045(20)30005-X 31928926

[113] Lin ZP Zhu YL Lo YC Moscarelli J Xiong A Korayem Y. Combination of triapine, olaparib, and cediranib suppresses progression of brca-wild type and parp inhibitor-resistant epithelial ovarian cancer. PLoS One. (2018) 13:e0207399. doi: 10.1371/journal.pone.0207399 30444904 PMC6239325

[114] Mirza MR Åvall Lundqvist E Birrer MJ dePont Christensen R Nyvang GB Malander S. Niraparib plus bevacizumab versus niraparib alone for platinum-sensitive recurrent ovarian cancer (Nsgo-avanova2/engot-ov24): A randomised, phase 2, superiority trial. Lancet Oncol. (2019) 20:1409–19. doi: 10.1016/S1470-2045(19)30515-7 31474354

[115] Ray-Coquard I Pautier P Pignata S Pérol D González-Martín A Berger R. Olaparib plus bevacizumab as first-line maintenance in ovarian cancer. New Engl J Med. (2019) 381:2416–28. doi: 10.1056/NEJMoa1911361 31851799

[116] Liu JF Barry WT Birrer M Lee JM Buckanovich RJ Fleming GF. Overall survival and updated progression-free survival outcomes in a randomized phase ii study of combination cediranib and olaparib versus olaparib in relapsed platinum-sensitive ovarian cancer. Ann oncology: Off J Eur Soc Med Oncol. (2019) 30:551–7. doi: 10.1093/annonc/mdz018 PMC6503628 30753272

[117] Liu JF Brady MF Matulonis UA Miller A Kohn EC Swisher EM. Olaparib with or without cediranib versus platinum-based chemotherapy in recurrent platinum-sensitive ovarian cancer (Nrg-gy004): A randomized, open-label, phase iii trial. J Clin oncology: Off J Am Soc Clin Oncol. (2022) 40:2138–47. doi: 10.1200/JCO.21.02011 PMC9242406 35290101

[118] Li Y Liu Y Zhang D Chen J Yang G Tang P. Discovery, synthesis, and evaluation of novel dual inhibitors of a vascular endothelial growth factor receptor and poly(Adp-ribose) polymerase for brca wild-type breast cancer therapy. J medicinal Chem. (2023) 66:12069–100. doi: 10.1021/acs.jmedchem.3c00640 37616488

[119] Schnekenburger M Dicato M Diederich MF. Anticancer potential of naturally occurring immunoepigenetic modulators: A promising avenue? Cancer. (2019) 125:1612–28. doi: 10.1002/cncr.32041 30840315

[120] Prorok P Forouzanfar F Murugarren N Peiffer I Charton R Akerman I. Loss of ezh2 function remodels the DNA replication initiation landscape. Cell Rep. (2023) 42:112280. doi: 10.1016/j.celrep.2023.112280 36995935

[121] Gao J Fosbrook C Gibson J Underwood TJ Gray JC Walters ZS. Review: targeting ezh2 in neuroblastoma. Cancer Treat Rev. (2023) 119:102600. doi: 10.1016/j.ctrv.2023.102600 37467626

[122] Xia J Li J Tian L Ren X Liu C Liang C. Targeting enhancer of zeste homolog 2 for the treatment of hematological Malignancies and solid tumors: candidate structure-activity relationships insights and evolution prospects. J medicinal Chem. (2022) 65:7016–43. doi: 10.1021/acs.jmedchem.2c00047 35531606

[123] Li X Wang C Li S Yin F Luo H Zhang Y. Dual target parp1/ezh2 inhibitors inducing excessive autophagy and producing synthetic lethality for triple-negative breast cancer therapy. Eur J Med Chem. (2024) 265:116054. doi: 10.1016/j.ejmech.2023.116054 38134746

[124] Kim KH Roberts CW. Targeting ezh2 in cancer. Nat Med. (2016) 22:128–34. doi: 10.1038/nm.4036 PMC4918227 26845405

[125] Souroullas GP Jeck WR Parker JS Simon JM Liu JY Paulk J. An oncogenic ezh2 mutation induces tumors through global redistribution of histone 3 lysine 27 trimethylation. Nat Med. (2016) 22:632–40. doi: 10.1038/nm.4092 PMC4899144 27135738

[126] Yu X Wang J Gong W Ma A Shen Y Zhang C. Dissecting and targeting noncanonical functions of ezh2 in multiple myeloma via an ezh2 degrader. Oncogene. (2023) 42:994–1009. doi: 10.1038/s41388-023-02618-5 36747009 PMC10040430

[127] Zhang X Huo X Guo H Xue L. Combined inhibition of parp and ezh2 for cancer treatment: current status, opportunities, and challenges. Front Pharmacol. (2022) 13:965244. doi: 10.3389/fphar.2022.965244 36263120 PMC9574044

[128] Yomtoubian S Lee SB Verma A Izzo F Markowitz G Choi H. Inhibition of ezh2 catalytic activity selectively targets a metastatic subpopulation in triple-negative breast cancer. Cell Rep. (2020) 30:755–70.e6. doi: 10.1016/j.celrep.2019.12.056 31968251

[129] Lee ST Li Z Wu Z Aau M Guan P Karuturi RK. Context-specific regulation of nf-Κb target gene expression by ezh2 in breast cancers. Mol Cell. (2011) 43:798–810. doi: 10.1016/j.molcel.2011.08.011 21884980

[130] Gonzalez ME Moore HM Li X Toy KA Huang W Sabel MS. Ezh2 expands breast stem cells through activation of notch1 signaling. Proc Natl Acad Sci United States America. (2014) 111:3098–103. doi: 10.1073/pnas.1308953111 PMC3939892 24516139

[131] Chen J Hong JH Huang Y Liu S Yin J Deng P. Ezh2 mediated metabolic rewiring promotes tumor growth independently of histone methyltransferase activity in ovarian cancer. Mol Cancer. (2023) 22:85. doi: 10.1186/s12943-023-01786-y 37210576 PMC10199584

[132] Yang AY Choi EB So Park M Kim SK Park MS Kim MY. Parp1 and prc2 double deficiency promotes brca-proficient breast cancer growth by modification of the tumor microenvironment. FEBS J. (2021) 288:2888–910. doi: 10.1111/febs.15636 33205541

[133] McCabe MT Ott HM Ganji G Korenchuk S Thompson C Van Aller GS. Ezh2 inhibition as a therapeutic strategy for lymphoma with ezh2-activating mutations. Nature. (2012) 492:108–12. doi: 10.1038/nature11606 23051747

[134] Hoy SM. Tazemetostat: first approval. Drugs. (2020) 80:513–21. doi: 10.1007/s40265-020-01288-x 32166598

[135] Wang C Qu L Li S Yin F Ji L Peng W. Discovery of first-in-class dual parp and ezh2 inhibitors for triple-negative breast cancer with wild-type brca. J medicinal Chem. (2021) 64:12630–50. doi: 10.1021/acs.jmedchem.1c00567 34455779

[136] Biersack B Polat S Höpfner M. Anticancer properties of chimeric hdac and kinase inhibitors. Semin Cancer Biol. (2022) 83:472–86. doi: 10.1016/j.semcancer.2020.11.005 33189849

[137] Kim IA Kim JH Shin JH Kim IH Kim JS Wu HG. A histone deacetylase inhibitor, trichostatin a, enhances radiosensitivity by abrogating G2/M arrest in human carcinoma cells. Cancer Res Treat. (2005) 37:122–8. doi: 10.4143/crt.2005.37.2.122 PMC2785402 19956491

[138] Liszczak G Diehl KL Dann GP Muir TW. Acetylation blocks DNA damage-induced chromatin adp-ribosylation. Nat Chem Biol. (2018) 14:837–40. doi: 10.1038/s41589-018-0097-1 PMC6505472 30013063

[139] Yin L Liu Y Peng Y Peng Y Yu X Gao Y. Parp inhibitor veliparib and hdac inhibitor saha synergistically co-target the uhrf1/brca1 DNA damage repair complex in prostate cancer cells. J Exp Clin Cancer research: CR. (2018) 37:153. doi: 10.1186/s13046-018-0810-7 30012171 PMC6048811

[140] Curtin NJ Szabo C. Therapeutic applications of parp inhibitors: anticancer therapy and beyond. Mol aspects Med. (2013) 34:1217–56. doi: 10.1016/j.mam.2013.01.006 PMC3657315 23370117

[141] Tangutoori S Baldwin P Sridhar S. Parp inhibitors: A new era of targeted therapy. Maturitas. (2015) 81:5–9. doi: 10.1016/j.maturitas.2015.01.015 25708226

[142] Zhang L Han Y Jiang Q Wang C Chen X Li X. Trend of histone deacetylase inhibitors in cancer therapy: isoform selectivity or multitargeted strategy. Medicinal Res Rev. (2015) 35:63–84. doi: 10.1002/med.21320 24782318

[143] Ha K Fiskus W Choi DS Bhaskara S Cerchietti L Devaraj SG. Histone deacetylase inhibitor treatment induces 'Brcaness' and synergistic lethality with parp inhibitor and cisplatin against human triple negative breast cancer cells. Oncotarget. (2014) 5:5637–50. doi: 10.18632/oncotarget.v5i14 PMC4170637 25026298

[144] Marijon H Lee DH Ding L Sun H Gery S de Gramont A. Co-targeting poly(Adp-ribose) polymerase (Parp) and histone deacetylase (Hdac) in triple-negative breast cancer: higher synergism in brca mutated cells. Biomedicine pharmacotherapy = Biomedecine pharmacotherapie. (2018) 99:543–51. doi: 10.1016/j.biopha.2018.01.045 29902865

[145] Valdez BC Nieto Y Yuan B Murray D Andersson BS. Hdac inhibitors suppress protein poly(Adp-ribosyl)Ation and DNA repair protein levels and phosphorylation status in hematologic cancer cells: implications for their use in combination with parp inhibitors and chemotherapeutic drugs. Oncotarget. (2022) 13:1122–35. doi: 10.18632/oncotarget.v13 PMC9564514 36243940

[146] Yuan Z Chen S Sun Q Wang N Li D Miao S. Olaparib hydroxamic acid derivatives as dual parp and hdac inhibitors for cancer therapy. Bioorg Med Chem. (2017) 25:4100–9. doi: 10.1016/j.bmc.2017.05.058 28601509

[147] Sdelci S Rendeiro AF Rathert P You W Lin JG Ringler A. Mthfd1 interaction with brd4 links folate metabolism to transcriptional regulation. Nat Genet. (2019) 51:990–8. doi: 10.1038/s41588-019-0413-z PMC6952269 31133746

[148] Filippakopoulos P Picaud S Mangos M Keates T Lambert JP Barsyte-Lovejoy D. Histone recognition and large-scale structural analysis of the human bromodomain family. Cell. (2012) 149:214–31. doi: 10.1016/j.cell.2012.02.013 PMC3326523 22464331

[149] Donati B Lorenzini E Ciarrocchi A. Brd4 and cancer: going beyond transcriptional regulation. Mol Cancer. (2018) 17:164. doi: 10.1186/s12943-018-0915-9 30466442 PMC6251205

[150] Qian H Zhu M Tan X Zhang Y Liu X Yang L. Super-enhancers and the super-enhancer reader brd4: tumorigenic factors and therapeutic targets. Cell Death Discovery. (2023) 9:470. doi: 10.1038/s41420-023-01775-6 38135679 PMC10746725

[151] Lu X Peng L Ding J Li Y Li Q Rao M. A deregulated M(6)a writer complex axis driven by brd4 confers an epitranscriptomic vulnerability in combined DNA repair-targeted therapy. Proc Natl Acad Sci United States America. (2023) 120:e2304534120. doi: 10.1073/pnas.2304534120 PMC10576145 37782793

[152] Sun C Yin J Fang Y Chen J Jeong KJ Chen X. Brd4 inhibition is synthetic lethal with parp inhibitors through the induction of homologous recombination deficiency. Cancer Cell. (2018) 33:401–16.e8. doi: 10.1016/j.ccell.2018.01.019 29533782 PMC5944839

[153] Yang L Zhang Y Shan W Hu Z Yuan J Pi J. Repression of bet activity sensitizes homologous recombination-proficient cancers to parp inhibition. Sci Trans Med. (2017) 9:eaal1645. doi: 10.1126/scitranslmed.aal1645 PMC5705017 28747513

[154] Filippakopoulos P Qi J Picaud S Shen Y Smith WB Fedorov O. Selective inhibition of bet bromodomains. Nature. (2010) 468:1067–73. doi: 10.1038/nature09504 PMC3010259 20871596

[155] Karakashev S Zhu H Yokoyama Y Zhao B Fatkhutdinov N Kossenkov AV. Bet bromodomain inhibition synergizes with parp inhibitor in epithelial ovarian cancer. Cell Rep. (2017) 21:3398–405. doi: 10.1016/j.celrep.2017.11.095 PMC5745042 29262321

[156] Mio C Gerratana L Bolis M Caponnetto F Zanello A Barbina M. Bet proteins regulate homologous recombination-mediated DNA repair: brcaness and implications for cancer therapy. Int J Cancer. (2019) 144:755–66. doi: 10.1002/ijc.31898 30259975

[157] Wang SP Li Y Huang SH Wu SQ Gao LL Sun Q. Discovery of potent and novel dual parp/brd4 inhibitors for efficient treatment of pancreatic cancer. J medicinal Chem. (2021) 64:17413–35. doi: 10.1021/acs.jmedchem.1c01535 34813314

[158] Zhang J Yang C Tang P Chen J Zhang D Li Y. Discovery of 4-hydroxyquinazoline derivatives as small molecular bet/parp1 inhibitors that induce defective homologous recombination and lead to synthetic lethality for triple-negative breast cancer therapy. J medicinal Chem. (2022) 65:6803–25. doi: 10.1021/acs.jmedchem.2c00135 35442700

[159] Elmenier FM Lasheen DS Abouzid KAM. Phosphatidylinositol 3 kinase (Pi3k) inhibitors as new weapon to combat cancer. Eur J Med Chem. (2019) 183:111718. doi: 10.1016/j.ejmech.2019.111718 31581005

[160] Fruman DA Chiu H Hopkins BD Bagrodia S Cantley LC Abraham RT. The pi3k pathway in human disease. Cell. (2017) 170:605–35. doi: 10.1016/j.cell.2017.07.029 PMC5726441 28802037

[161] Chandarlapaty S Sawai A Scaltriti M Rodrik-Outmezguine V Grbovic-Huezo O Serra V. Akt inhibition relieves feedback suppression of receptor tyrosine kinase expression and activity. Cancer Cell. (2011) 19:58–71. doi: 10.1016/j.ccr.2010.10.031 21215704 PMC3025058

[162] Serra V Scaltriti M Prudkin L Eichhorn PJ Ibrahim YH Chandarlapaty S. Pi3k inhibition results in enhanced her signaling and acquired erk dependency in her2-overexpressing breast cancer. Oncogene. (2011) 30:2547–57. doi: 10.1038/onc.2010.626 PMC3107390 21278786

[163] Ibrahim YH García-García C Serra V He L Torres-Lockhart K Prat A. Pi3k inhibition impairs brca1/2 expression and sensitizes brca-proficient triple-negative breast cancer to parp inhibition. Cancer Discovery. (2012) 2:1036–47. doi: 10.1158/2159-8290.CD-11-0348 PMC5125254 22915752

[164] Chalmers AJ Lakshman M Chan N Bristow RG. Poly(Adp-ribose) polymerase inhibition as a model for synthetic lethality in developing radiation oncology targets. Semin Radiat Oncol. (2010) 20:274–81. doi: 10.1016/j.semradonc.2010.06.001 20832020

[165] Jang NY Kim DH Cho BJ Choi EJ Lee JS Wu HG. Radiosensitization with combined use of olaparib and pi-103 in triple-negative breast cancer. BMC Cancer. (2015) 15:89. doi: 10.1186/s12885-015-1090-7 25884663 PMC4355140

[166] Cardnell RJ Feng Y Mukherjee S Diao L Tong P Stewart CA. Activation of the pi3k/mtor pathway following parp inhibition in small cell lung cancer. PLoS One. (2016) 11:e0152584. doi: 10.1371/journal.pone.0152584 27055253 PMC4824499

[167] Juvekar A Burga LN Hu H Lunsford EP Ibrahim YH Balmañà J. Combining a pi3k inhibitor with a parp inhibitor provides an effective therapy for brca1-related breast cancer. Cancer Discovery. (2012) 2:1048–63. doi: 10.1158/2159-8290.CD-11-0336 PMC3733368 22915751

[168] Bian X Gao J Luo F Rui C Zheng T Wang D. Pten deficiency sensitizes endometrioid endometrial cancer to compound parp-pi3k inhibition but not parp inhibition as monotherapy. Oncogene. (2018) 37:341–51. doi: 10.1038/onc.2017.326 PMC5799770 28945226

[169] Li Y Wang Y Zhang W Wang X Chen L Wang S. Bkm120 sensitizes brca-proficient triple negative breast cancer cells to olaparib through regulating foxm1 and exo1 expression. Sci Rep. (2021) 11:4774. doi: 10.1038/s41598-021-82990-y 33637776 PMC7910492

[170] Matulonis UA Wulf GM Barry WT Birrer M Westin SN Farooq S. Phase I dose escalation study of the pi3kinase pathway inhibitor bkm120 and the oral poly (Adp ribose) polymerase (Parp) inhibitor olaparib for the treatment of high-grade serous ovarian and breast cancer. Ann oncology: Off J Eur Soc Med Oncol. (2017) 28:512–8. doi: 10.1093/annonc/mdw672 PMC5834157 27993796

[171] Batalini F Xiong N Tayob N Polak M Eismann J Cantley LC. Phase 1b clinical trial with alpelisib plus olaparib for patients with advanced triple-negative breast cancer. Clin Cancer research: an Off J Am Assoc Cancer Res. (2022) 28:1493–9. doi: 10.1158/1078-0432.CCR-21-3045 PMC9066379 35149538

[172] Wang J Li H He G Chu Z Peng K Ge Y. Discovery of novel dual poly(Adp-ribose)Polymerase and phosphoinositide 3-kinase inhibitors as a promising strategy for cancer therapy. J medicinal Chem. (2020) 63:122–39. doi: 10.1021/acs.jmedchem.9b00622 31846325

[173] Wang J He G Li H Ge Y Wang S Xu Y. Discovery of novel parp/pi3k dual inhibitors with high efficiency against brca-proficient triple negative breast cancer. Eur J Med Chem. (2021) 213:113054. doi: 10.1016/j.ejmech.2020.113054 33309164

[174] Wu Z Bai Y Jin J Jiang T Shen H Ju Q. Discovery of novel and potent parp/pi3k dual inhibitors for the treatment of cancer. Eur J Med Chem. (2021) 217:113357. doi: 10.1016/j.ejmech.2021.113357 33740547

[175] Ors A Chitsazan AD Doe AR Mulqueen RM Ak C Wen Y. Estrogen regulates divergent transcriptional and epigenetic cell states in breast cancer. Nucleic Acids Res. (2022) 50:11492–508. doi: 10.1093/nar/gkac908 PMC9723652 36318267

[176] Siersbæk R Scabia V Nagarajan S Chernukhin I Papachristou EK Broome R. Il6/stat3 signaling hijacks estrogen receptor Α Enhancers to drive breast cancer metastasis. Cancer Cell. (2020) 38:412–23.e9. doi: 10.1016/j.ccell.2020.06.007 32679107 PMC7116707

[177] Patel S Homaei A Raju AB Meher BR. Estrogen: the necessary evil for human health, and ways to tame it. Biomedicine pharmacotherapy = Biomedecine pharmacotherapie. (2018) 102:403–11. doi: 10.1016/j.biopha.2018.03.078 29573619

[178] Maggi A. Liganded and unliganded activation of estrogen receptor and hormone replacement therapies. Biochim Biophys Acta. (2011) 1812:1054–60. doi: 10.1016/j.bbadis.2011.05.001 PMC3121051 21605666

[179] Mueller CR Roskelley CD. Regulation of brca1 expression and its relationship to sporadic breast cancer. Breast Cancer research: BCR. (2003) 5:45–52. doi: 10.1186/bcr557 12559046 PMC154136

[180] Marquis ST Rajan JV Wynshaw-Boris A Xu J Yin GY Abel KJ. The developmental pattern of brca1 expression implies a role in differentiation of the breast and other tissues. Nat Genet. (1995) 11:17–26. doi: 10.1038/ng0995-17 7550308

[181] Fan S Wang J Yuan R Ma Y Meng Q Erdos MR. Brca1 inhibition of estrogen receptor signaling in transfected cells. Sci (New York NY). (1999) 284:1354–6. doi: 10.1126/science.284.5418.1354 10334989

[182] Fan S Ma YX Wang C Yuan RQ Meng Q Wang JA. Role of direct interaction in brca1 inhibition of estrogen receptor activity. Oncogene. (2001) 20:77–87. doi: 10.1038/sj.onc.1204073 11244506

[183] Hu Y Ghosh S Amleh A Yue W Lu Y Katz A. Modulation of aromatase expression by brca1: A possible link to tissue-specific tumor suppression. Oncogene. (2005) 24:8343–8. doi: 10.1038/sj.onc.1208985 16170371

[184] Zhou S Liu Y Jin L Guo P Liu Q Shan J. Estrogen enhances the cytotoxicity of parp inhibitors on breast cancer cells through stimulating nitric oxide production. J Steroid Biochem Mol Biol. (2021) 209:105853. doi: 10.1016/j.jsbmb.2021.105853 33617965

[185] Traphagen NA Schwartz GN Tau S Roberts AM Jiang A Hosford SR. Estrogen therapy induces receptor-dependent DNA damage enhanced by parp inhibition in er+ Breast cancer. Clin Cancer research: an Off J Am Assoc Cancer Res. (2023) 29:3717–28. doi: 10.1158/1078-0432.CCR-23-0488 PMC10528687 37439680

[186] Riley JL. Pd-1 signaling in primary T cells. Immunol Rev. (2009) 229:114–25. doi: 10.1111/j.1600-065X.2009.00767.x PMC3424066 19426218

[187] Paterson AM Brown KE Keir ME Vanguri VK Riella LV Chandraker A. The programmed death-1 ligand 1:B7-1 pathway restrains diabetogenic effector T cells in vivo. J Immunol (Baltimore Md: 1950). (2011) 187:1097–105. doi: 10.4049/jimmunol.1003496 PMC3148082 21697456

[188] Daassi D Mahoney KM Freeman GJ. The importance of exosomal pdl1 in tumour immune evasion. Nat Rev Immunol. (2020) 20:209–15. doi: 10.1038/s41577-019-0264-y 31965064

[189] Jiao S Xia W Yamaguchi H Wei Y Chen MK Hsu JM. Parp inhibitor upregulates pd-L1 expression and enhances cancer-associated immunosuppression. Clin Cancer research: an Off J Am Assoc Cancer Res. (2017) 23:3711–20. doi: 10.1158/1078-0432.CCR-16-3215 PMC5511572 28167507

[190] Sato H Niimi A Yasuhara T Permata TBM Hagiwara Y Isono M. DNA double-strand break repair pathway regulates pd-L1 expression in cancer cells. Nat Commun. (2017) 8:1751. doi: 10.1038/s41467-017-01883-9 29170499 PMC5701012

[191] Wu M Huang Q Xie Y Wu X Ma H Zhang Y. Improvement of the anticancer efficacy of pd-1/pd-L1 blockade via combination therapy and pd-L1 regulation. J Hematol Oncol. (2022) 15:24. doi: 10.1186/s13045-022-01242-2 35279217 PMC8917703

[192] Kornepati AVR Boyd JT Murray CE Saifetiarova J de la Peña Avalos B Rogers CM. Tumor intrinsic pd-L1 promotes DNA repair in distinct cancers and suppresses parp inhibitor-induced synthetic lethality. Cancer Res. (2022) 82:2156–70. doi: 10.1158/0008-5472.CAN-21-2076 PMC9987177 35247877

[193] Bound NT Vandenberg CJ Kartikasari AER Plebanski M Scott CL. Improving parp inhibitor efficacy in high-grade serous ovarian carcinoma: A focus on the immune system. Front Genet. (2022) 13:886170. doi: 10.3389/fgene.2022.886170 36159999 PMC9505691

[194] Pantelidou C Sonzogni O De Oliveria Taveira M Mehta AK Kothari A Wang D. Parp inhibitor efficacy depends on cd8(+) T-cell recruitment via intratumoral sting pathway activation in brca-deficient models of triple-negative breast cancer. Cancer Discovery. (2019) 9:722–37. doi: 10.1158/2159-8290.CD-18-1218 PMC6548644 31015319

[195] Ding L Kim HJ Wang Q Kearns M Jiang T Ohlson CE. Parp inhibition elicits sting-dependent antitumor immunity in brca1-deficient ovarian cancer. Cell Rep. (2018) 25:2972–80.e5. doi: 10.1016/j.celrep.2018.11.054 30540933 PMC6366450

[196] Bretscher PA. A two-step, two-signal model for the primary activation of precursor helper T cells. Proc Natl Acad Sci United States America. (1999) 96:185–90. doi: 10.1073/pnas.96.1.185 PMC15114 9874793

[197] Chabanon RM Muirhead G Krastev DB Adam J Morel D Garrido M. Parp inhibition enhances tumor cell-intrinsic immunity in ercc1-deficient non-small cell lung cancer. J Clin Invest. (2019) 129:1211–28. doi: 10.1172/JCI123319 PMC6391116 30589644

[198] Sen T Rodriguez BL Chen L Corte CMD Morikawa N Fujimoto J. Targeting DNA damage response promotes antitumor immunity through sting-mediated T-cell activation in small cell lung cancer. Cancer Discovery. (2019) 9:646–61. doi: 10.1158/2159-8290.CD-18-1020 PMC6563834 30777870

[199] Shen J Zhao W Ju Z Wang L Peng Y Labrie M. Parpi triggers the sting-dependent immune response and enhances the therapeutic efficacy of immune checkpoint blockade independent of brcaness. Cancer Res. (2019) 79:311–9. doi: 10.1158/0008-5472.CAN-18-1003 PMC6588002 30482774

[200] Wang Z Sun K Xiao Y Feng B Mikule K Ma X. Niraparib activates interferon signaling and potentiates anti-pd-1 antibody efficacy in tumor models. Sci Rep. (2019) 9:1853. doi: 10.1038/s41598-019-38534-6 30755715 PMC6372650

[201] Lampert EJ Zimmer A Padget M Cimino-Mathews A Nair JR Liu Y. Combination of parp inhibitor olaparib, and pd-L1 inhibitor durvalumab, in recurrent ovarian cancer: A proof-of-concept phase ii study. Clin Cancer research: an Off J Am Assoc Cancer Res. (2020) 26:4268–79. doi: 10.1158/1078-0432.CCR-20-0056 PMC7442720 32398324

[202] Tan TJ Sammons S Im YH She L Mundy K Bigelow R. Phase ii dora study of olaparib with or without durvalumab as a chemotherapy-free maintenance strategy in platinum-pretreated advanced triple-negative breast cancer. Clin Cancer research: an Off J Am Assoc Cancer Res. (2024) 30:1240–7. doi: 10.1158/1078-0432.CCR-23-2513 PMC10982642 38236575

[203] Konstantinopoulos PA Waggoner S Vidal GA Mita M Moroney JW Holloway R. Single-arm phases 1 and 2 trial of niraparib in combination with pembrolizumab in patients with recurrent platinum-resistant ovarian carcinoma. JAMA Oncol. (2019) 5:1141–9. doi: 10.1001/jamaoncol.2019.1048 PMC6567832 31194228

[204] Mutch D Voulgari A Chen XM Bradley WH Oaknin A Perez Fidalgo JA. Primary results and characterization of patients with exceptional outcomes in a phase 1b study combining parp and mek inhibition, with or without anti-pd-L1, for brca wild-type, platinum-sensitive, recurrent ovarian cancer. Cancer. (2024) 130:1940–51. doi: 10.1002/cncr.35222 38288862

[205] Swisher EM Lin KK Oza AM Scott CL Giordano H Sun J. Rucaparib in relapsed, platinum-sensitive high-grade ovarian carcinoma (Ariel2 part 1): an international, multicentre, open-label, phase 2 trial. Lancet Oncol. (2017) 18:75–87. doi: 10.1016/S1470-2045(16)30559-9 27908594

[206] Banerjee S Imbimbo M Roxburgh P Kim J Kim MH Plummer R. 529mo phase ii study of olaparib plus durvalumab with or without bevacizumab (Mediola): final analysis of overall survival in patients with non-germline brca-mutated platinum-sensitive relapsed ovarian cancer. Ann Oncol. (2022). doi: 10.1016/j.annonc.2022.07.657

[207] Yan M Wu S Wang Y Liang M Wang M Hu W. Recent progress of supramolecular chemotherapy based on host-guest interactions. Advanced materials (Deerfield Beach Fla). (2023) 36:e2304249. doi: 10.1002/adma.202304249 37478832

[208] Hossain MB Haldar Neer AH. Chemotherapy. Cancer Treat Res. (2023) 185:49–58. doi: 10.1007/978-3-031-27156-4_3 37306903

[209] Lu Y Liu Y Pang Y Pacak K Yang C. Double-barreled gun: combination of parp inhibitor with conventional chemotherapy. Pharmacol Ther. (2018) 188:168–75. doi: 10.1016/j.pharmthera.2018.03.006 PMC6067963 29621593

[210] Loibl S O'Shaughnessy J Untch M Sikov WM Rugo HS McKee MD. Addition of the parp inhibitor veliparib plus carboplatin or carboplatin alone to standard neoadjuvant chemotherapy in triple-negative breast cancer (Brightness): A randomised, phase 3 trial. Lancet Oncol. (2018) 19:497–509. doi: 10.1016/S1470-2045(18)30111-6 29501363

[211] Swisher EM Aghajanian C O'Malley DM Fleming GF Kaufmann SH Levine DA. Impact of homologous recombination status and responses with veliparib combined with first-line chemotherapy in ovarian cancer in the phase 3 velia/gog-3005 study. Gynecol Oncol. (2022) 164:245–53. doi: 10.1016/j.ygyno.2021.12.003 34906376

[212] Aghajanian C Swisher EM Okamoto A Steffensen KD Bookman MA Fleming GF. Impact of veliparib, paclitaxel dosing regimen, and germline brca status on the primary treatment of serous ovarian cancer - an ancillary data analysis of the velia trial. Gynecol Oncol. (2022) 164:278–87. doi: 10.1016/j.ygyno.2021.12.012 PMC9399938 34930617

[213] Rodler E Sharma P Barlow WE Gralow JR Puhalla SL Anders CK. Cisplatin with veliparib or placebo in metastatic triple-negative breast cancer and brca mutation-associated breast cancer (S1416): A randomised, double-blind, placebo-controlled, phase 2 trial. Lancet Oncol. (2023) 24:162–74. doi: 10.1016/S1470-2045(22)00739-2 PMC9924094 36623515

[214] Tariq MR Ali SW Fatima N Jabeen A Qazi AS Hameed A. Radiation therapies in cancer. Cancer Treat Res. (2023) 185:59–77. doi: 10.1007/978-3-031-27156-4_4 37306904

[215] Lawhn-Heath C Hope TA Martinez J Fung EK Shin J Seo Y. Dosimetry in radionuclide therapy: the clinical role of measuring radiation dose. Lancet Oncol. (2022) 23:e75–87. doi: 10.1016/S1470-2045(21)00657-4 35114134

[216] Goodhead DT. Initial events in the cellular effects of ionizing radiations: clustered damage in DNA. Int J Radiat Biol. (1994) 65:7–17. doi: 10.1080/09553009414550021 7905912

[217] Ranjha L Howard SM Cejka P. Main steps in DNA double-strand break repair: an introduction to homologous recombination and related processes. Chromosoma. (2018) 127:187–214. doi: 10.1007/s00412-017-0658-1 29327130

[218] Otazo R Lambin P Pignol JP Ladd ME Schlemmer HP Baumann M. Mri-guided radiation therapy: An emerging paradigm in adaptive radiation oncology. Radiology. (2021) 298:248–60. doi: 10.1148/radiol.2020202747 PMC7924409 33350894

[219] Boussios S Rassy E Moschetta M Ghose A Adeleke S Sanchez E. Brca mutations in ovarian and prostate cancer: bench to bedside. Cancers. (2022) 14:3888. doi: 10.3390/cancers14163888 PMC9405840 36010882

[220] Huang RX Zhou PK. DNA damage response signaling pathways and targets for radiotherapy sensitization in cancer. Signal transduction targeted Ther. (2020) 5:60. doi: 10.1038/s41392-020-0150-x PMC7192953 32355263

[221] Loap P Loirat D Berger F Rodrigues M Bazire L Pierga JY. Concurrent olaparib and radiotherapy in patients with triple-negative breast cancer: the phase 1 olaparib and radiation therapy for triple-negative breast cancer trial. JAMA Oncol. (2022) 8:1802–8. doi: 10.1001/jamaoncol.2022.5074 PMC9614672 36301572

[222] Deng S Vlatkovic T Li M Zhan T Veldwijk MR Herskind C. Targeting the DNA damage response and DNA repair pathways to enhance radiosensitivity in colorectal cancer. Cancers. (2022) 14:4874. doi: 10.3390/cancers14194874 PMC9561988 36230796

[223] Yang SH Kuo TC Wu H Guo JC Hsu C Hsu CH. Perspectives on the combination of radiotherapy and targeted therapy with DNA repair inhibitors in the treatment of pancreatic cancer. World J Gastroenterol. (2016) 22:7275–88. doi: 10.3748/wjg.v22.i32.7275 PMC4997635 27621574

[224] de Haan R van den Heuvel MM van Diessen J Peulen HMU van Werkhoven E de Langen AJ. Phase I and pharmacologic study of olaparib in combination with high-dose radiotherapy with and without concurrent cisplatin for non-small cell lung cancer. Clin Cancer research: an Off J Am Assoc Cancer Res. (2021) 27:1256–66. doi: 10.1158/1078-0432.CCR-20-2551 33262140

[225] Dungey FA Löser DA Chalmers AJ. Replication-dependent radiosensitization of human glioma cells by inhibition of poly(Adp-ribose) polymerase: mechanisms and therapeutic potential. Int J Radiat oncology biology Phys. (2008) 72:1188–97. doi: 10.1016/j.ijrobp.2008.07.031 18954712

[226] Rivero Belenchón I Congregado Ruiz CB Saez C Osman García I Medina López RA. Parp inhibitors and radiotherapy: A new combination for prostate cancer (Systematic review). Int J Mol Sci. (2023) 24:12978. doi: 10.3390/ijms241612978 PMC10455664 37629155

[227] Michmerhuizen AR Pesch AM Moubadder L Chandler BC Wilder-Romans K Cameron M. Parp1 inhibition radiosensitizes models of inflammatory breast cancer to ionizing radiation. Mol Cancer Ther. (2019) 18:2063–73. doi: 10.1158/1535-7163.MCT-19-0520 PMC6825563 31413177

[228] Césaire M Thariat J Candéias SM Stefan D Saintigny Y Chevalier F. Combining parp inhibition, radiation, and immunotherapy: A possible strategy to improve the treatment of cancer? Int J Mol Sci. (2018) 19:3793. doi: 10.3390/ijms19123793 PMC6321381 30487462

[229] Dulaney C Marcrom S Stanley J Yang ES. Poly(Adp-ribose) polymerase activity and inhibition in cancer. Semin Cell Dev Biol. (2017) 63:144–53. doi: 10.1016/j.semcdb.2017.01.007 28087320

[230] Powell C Mikropoulos C Kaye SB Nutting CM Bhide SA Newbold K. Pre-clinical and clinical evaluation of parp inhibitors as tumour-specific radiosensitisers. Cancer Treat Rev. (2010) 36:566–75. doi: 10.1016/j.ctrv.2010.03.003 20409643

[231] Senra JM Telfer BA Cherry KE McCrudden CM Hirst DG O'Connor MJ. Inhibition of parp-1 by olaparib (Azd2281) increases the radiosensitivity of a lung tumor xenograft. Mol Cancer Ther. (2011) 10:1949–58. doi: 10.1158/1535-7163.MCT-11-0278 PMC3192032 21825006

[232] Zhao W Hu H Mo Q Guan Y Li Y Du Y. Function and mechanism of combined parp-1 and brca genes in regulating the radiosensitivity of breast cancer cells. Int J Clin Exp Pathol. (2019) 12:3915–20. PMC6949771 31933782

[233] Parsels LA Engelke CG Parsels J Flanagan SA Zhang Q Tanska D. Combinatorial efficacy of olaparib with radiation and atr inhibitor requires parp1 protein in homologous recombination-proficient pancreatic cancer. Mol Cancer Ther. (2021) 20:263–73. doi: 10.1158/1535-7163.MCT-20-0365 PMC7867626 33268569

[234] A phase I/ii study of olaparib with entinostat in the treatment of recurrent, platinum-refractory or resistant, homologous recombination repair proficient ovarian, primary peritoneal, and fallopian tube cancers. (2019).

[235] A phase ib combination study of rucaparib (Co-338) and atezolizumab (Mpdl3280a) in participants with advanced gynecologic cancers and triple-negative breast cancer. (2017).

[236] Part 1: avanova1 - a phase I study to evaluate the safety and tolerability of bevacizumab-niraparib combination therapy and determine the recommended phase 2 dose (Rp2d) in women with platinum-sensitive epithelial ovarian, fallopian tube, or peritoneal cancer part 2: avanova2 - a two-arm, open-label, phase ii randomized study to evaluate the efficacy of niraparib versus niraparib-bevacizumab combination in women with platinum-sensitive epithelial ovarian, fallopian tube, or peritoneal cancer. (2015).

[237] A study of rucaparib as switch maintenance following platinum-based chemotherapy in patients with platinum-sensitive, high-grade serous or endometrioid epithelial ovarian, primary peritoneal or fallopian tube cancer (Ariel3). (2013).

[238] Phase I/ii study of cediranib and olaparib in combination for treatment of recurrent papillary-serous ovarian, fallopian tube, or peritoneal cancer or for treatment of recurrent triple-negative breast cancer. (2010).

[239] Randomized, double-blind, phase iii trial olaparib vs. Placebo patients with advanced figo stage iiib-iv high grade serious or endometrioid ovarian, fallopian tube, or peritoneal cancer treated standard first-line treatment. (2015).

[240] A phase iii study comparing single-agent olaparib or the combination of cediranib and olaparib to standard platinum-based chemotherapy in women with recurrent platinum-sensitive ovarian, fallopian tube, or primary peritoneal cancer. (2015).

[241] A single-arm phase ii study of olaparib maintenance with pembrolizumab & Bevacizumab in brca non-mutated patients with platinum-sensitive recurrent ovarian cancer (Opeb-01). (2020). 10.3802/jgo.2021.32.e31 PMC7930449 33559413

[242] Phase 1 trial of abt-888 and sch727965 in patients with advanced solid tumors. (2011).

[243] A randomized, molecular driven phase ii trial of carboplatin-paclitaxel-bevacizumab vs carboplatin-paclitaxel-bevacizumab-rucaparib vs carboplatin-paclitaxel-rucaparib, selected according to hrd status, in patients with advanced (Stage iii B-C-iv) ovarian, primary peritoneal and fallopian tube cancer preceded by a phase I dose escalation study on rucaparib-bevacizumab combination. (2018).

[244] A randomized phase 3, double-blind study of chemotherapy with or without pembrolizumab followed by maintenance with olaparib or placebo for the first-line treatment of brca non-mutated advanced epithelial ovarian cancer (Eoc) (Keylynk-001 / engot-ov43 / gog-3036). (2018).

[245] International phase iii randomised study to evaluate the efficacy of maintenance therapy with olaparib and cediranib or olaparib alone in patients with relapsed ovarian cancer following a response to platinum-based chemotherapy. (2017). 10.1136/ijgc-2020-002073 33097567

[246] A Phase Iii Randomized, Double-Blinded Trial of Platinum-Based Chemotherapy with or without Atezolizumab Followed by Niraparib Maintenance with or without Atezolizumab in Patients with Recurrent Ovarian, Tubal or Peritoneal Cancer and Platinum Treatment-Free Interval (Tfip) >6 Months. (2018). 10.1136/ijgc-2020-001633 33318079

